# Happiness Maximization Is a WEIRD Way of Living

**DOI:** 10.1177/17456916231208367

**Published:** 2024-02-13

**Authors:** Kuba Krys, Olga Kostoula, Wijnand A. P. van Tilburg, Oriana Mosca, J. Hannah Lee, Fridanna Maricchiolo, Aleksandra Kosiarczyk, Agata Kocimska-Bortnowska, Claudio Torres, Hidefumi Hitokoto, Kongmeng Liew, Michael H. Bond, Vivian Miu-Chi Lun, Vivian L. Vignoles, John M. Zelenski, Brian W. Haas, Joonha Park, Christin-Melanie Vauclair, Anna Kwiatkowska, Marta Roczniewska, Nina Witoszek, İdil Işık, Natasza Kosakowska-Berezecka, Alejandra Domínguez-Espinosa, June Chun Yeung, Maciej Górski, Mladen Adamovic, Isabelle Albert, Vassilis Pavlopoulos, Márta Fülöp, David Sirlopu, Ayu Okvitawanli, Diana Boer, Julien Teyssier, Arina Malyonova, Alin Gavreliuc, Ursula Serdarevich, Charity S. Akotia, Lily Appoh, D. M. Arévalo Mira, Arno Baltin, Patrick Denoux, Carla Sofia Esteves, Vladimer Gamsakhurdia, Ragna B. Garðarsdóttir, David O. Igbokwe, Eric R. Igou, Natalia Kascakova, Lucie Klůzová Kračmárová, Nicole Kronberger, Pablo Eduardo Barrientos, Tamara Mohorić, Elke Murdock, Nur Fariza Mustaffa, Martin Nader, Azar Nadi, Yvette van Osch, Zoran Pavlović, Iva Poláčková Šolcová, Muhammad Rizwan, Vladyslav Romashov, Espen Røysamb, Ruta Sargautyte, Beate Schwarz, Lenka Selecká, Heyla A. Selim, Maria Stogianni, Chien-Ru Sun, Agnieszka Wojtczuk-Turek, Cai Xing, Yukiko Uchida

**Affiliations:** 1 Institute of Psychology, Polish Academy of Sciences; 2 Institute of Psychology, Johannes Kepler University Linz; 3 Department of Psychology, University of Essex; 4 Department of Education, Psychology, and Philosophy, University of Cagliari; 5 Department of Psychology, Indiana University Northwest; 6 Department of Education, University of Roma Tre; 7 SWPS University; 8 Institute of Psychology, University of Brasilia; 9 Department of Psychological Sciences, Kwansei Gakuin University; 10 Graduate School of Human and Environmental Studies, Kyoto University; 11 School of Psychology, Speech and Hearing, University of Canterbury; 12 Department of Management and Marketing, Faculty of Business, Hong Kong Polytechnic University; 13 Department of Applied Psychology, Lingnan University; 14 School of Psychology, University of Sussex; 15 Department of Psychology, Carleton University; 16 Department of Psychology, University of Georgia; 17 Graduate School of Management, NUCB Business School; 18 Centre for Psychological Research and Social Intervention (CIS-Iscte), Iscte-Instituto Universitário de Lisboa; 19 Department of Learning, Informatics, Management and Ethics, Medical Management Centre, Karolinska Institutet; 20 Centre for Development and the Environment, University of Oslo; 21 Psychology Department, Bahçeşehir University; 22 Institute of Psychology, University of Gdansk; 23 Department of Psychology, Iberoamerican University; 24 Faculty of Psychology, University of Warsaw; 25 King’s Business School, King’s College London; 26 Department of Behavioural and Cognitive Sciences, University of Luxembourg; 27 Department of Psychology, National and Kapodistrian University of Athens; 28 Institute of Psychology, Károli Gáspár University of the Reformed Church; 29 Research Centre of Natural Sciences, Institute of Cognitive Neuroscience and Psychology, Eötvös Loránd Research Network, Budapest, Hungary; 30 Faculty of Psychology and Humanities, Universidad San Sebastián, Concepción; 31 Department of Psychology, Universitas Brawijaya; 32 Institute of Psychology, University of Koblenz; 33 Département Psychologie Clinique Du Sujet, Université Toulouse II; 34 Department of General and Social Psychology, Dostoevsky Omsk State University; 35 Department of Psychology, West University of Timisoara; 36 Universidad Nacional del Oeste; 37 Universidad National de Hurlingham; 38 Department of Psychology, School of Social Sciences, University of Ghana; 39 Faculty of Nursing and Health Sciences, Nord University; 40 HULAB, San Salvador, El Salvador; 41 School of Natural Sciences and Health, Tallinn University; 42 Universidade Católica Portuguesa, Católica Lisbon School of Business and Economics, Católica Lisbon Research Unit in Business and Economics; 43 Department of Psychology, Ivane Javakhishvili Tbilisi State University; 44 Faculty of Psychology, University of Iceland; 45 Baze University Abuja; 46 Department of Psychology, University of Limerick; 47 Olomouc University Social Health Institute, Palacky University; 48 Psychiatric Clinic Pro Mente Sana, Bratislava, Slovakia; 49 Institute of Psychology, Czech Academy of Sciences; 50 Department of Psychology, Universidad del Valle de Guatemala; 51 Department of Psychology, Faculty of Humanities and Social Sciences, University of Rijeka; 52 Department of Business Administration, International Islamic University Malaysia; 53 Department of Psychological Studies, Universidad ICESI; 54 Department of Social Psychology, Tilburg School of Social and Behavioral Sciences, Tilburg University; 55 Department of Psychology, Faculty of Philosophy University of Belgrade; 56 Department of Psychology, University of Haripur; 57 Department of Psychology, University of Oslo; 58 Institute of Psychology, Faculty of Philosophy, Vilnius University; 59 Department of Applied Psychology, Zurich University of Applied Sciences; 60 University of St. Cyril and Methodius of Trnava; 61 King Saud University; 62 Department of Culture Studies, Tilburg University; 63 Department of Psychology, National Chengchi University; 64 Warsaw School of Economics; 65 Department of Psychology, Renmin University of China; 66 Institute for the Future of Human Society, Kyoto University

**Keywords:** culture, society, subjective well-being, happiness, life satisfaction

## Abstract

Psychological science tends to treat subjective well-being and happiness synonymously. We start from the assumption that subjective well-being is more than being happy to ask the fundamental question: What is the *ideal* level of happiness? From a cross-cultural perspective, we propose that the idealization of attaining maximum levels of happiness may be especially characteristic of Western, educated, industrial, rich, and democratic (WEIRD) societies but less so for others. Searching for an explanation for why “happiness maximization” might have emerged in these societies, we turn to studies linking cultures to their eco-environmental habitat. We discuss the premise that WEIRD cultures emerged in an exceptionally benign ecological habitat (i.e., faced relatively light existential pressures compared with other regions). We review the influence of the Gulf Stream on the Northwestern European climate as a source of these comparatively benign geographical conditions. We propose that the ecological conditions in which WEIRD societies emerged afforded them a basis to endorse happiness as a value and to idealize attaining its maximum level. To provide a nomological network for happiness maximization, we also studied some of its potential side effects, namely alcohol and drug consumption and abuse and the prevalence of mania. To evaluate our hypothesis, we reanalyze data from two large-scale studies on ideal levels of personal life satisfaction—the most common operationalization of happiness in psychology—involving respondents from 61 countries. We conclude that societies whose members seek to maximize happiness tend to be characterized as WEIRD, and generalizing this across societies can prove problematic if adopted at the ideological and policy level.

Happiness isn’t good enough for me. I demand euphoria!!!

—Calvin talking in “Calvin and Hobbes” by Bill Watterson

Umm . . . I never thought about such a thing in my whole life.

—Japanese adult, asked whether he is happy (from field studies of Hidefumi Hitokoto)

Psychological science generally understands happiness as a sense of satisfaction combined with the presence of positive feelings and absence of negative feelings (Kim-Prieto et al., 2005). It treats happiness as tantamount to subjective well-being. However, inquiring about people’s desired levels of happiness may reveal a distinction between the two. Specifically, the possibility that people seek moderate rather than extensive levels of happiness would suggest that subjective well-being and happiness are not interchangeable. How important do people across the globe find it to be happy? Do people universally wish to maximize their happiness, or do people in some societies prefer intermediate levels? How important is happiness relative to meaning, spirituality, and harmony, and what cultural or ecological factors might influence their relative prioritization?

The interchangeability of happiness and subjective well-being is a hallmark feature of the majority of contemporary subjective well-being and happiness research, especially that within the framework of Diener and colleagues’ (1995) theory of subjective well-being. The empirical basis of Diener’s theory originates primarily from Westernized, educated, industrialized, rich, and democratic (WEIRD) societies (Henrich et al., 2010). Accordingly, insights into the structure of subjective well-being, the importance of happiness relative to other components of subjective well-being, and the degree of happiness pursued by groups and their individual members reflects views that may not necessarily generalize to other societies. Although Diener and colleagues’ model has been tremendously helpful in guiding the study of subjective well-being and happiness and raising their profile as variables of interest to psychologists and policymakers, we contend that it is time to reevaluate some basic assumptions of subjective well-being research in light of emerging findings in cross-cultural psychology.

First, we examine the differentiation between subjective well-being and happiness. Many theoretical and empirical treatises of subjective well-being equate it with happiness, and this arguably represents the dominant view in psychological literature (e.g., “The empirical science of subjective wellbeing, popularly referred to as happiness or satisfaction. . .” on p. 253 in Diener et al., 2018; see also Das et al., 2020; Dolan et al., 2008; Luhmann, 2017; Schimmack, 2006; cf. Flanagan et al., 2023; Joshanloo et al., 2021). We contend that subjective well-being is more than being happy in many societies. Treating happiness as tantamount to subjective well-being may represent too narrow an understanding of subjective well-being that overlooks other potentially important components. To address this problem, we propose a broader model of subjective well-being that recognizes happiness as one of several interdependent components constituting subjective well-being.

Second, we suggest that the cultural variation in ideal levels of happiness warrants theoretical integration into contemporary models of subjective well-being. We review research that highlights important cultural differences in the positioning of happiness as a superordinate outcome among various components of subjective well-being. The resultant implication is that people seek to increase their levels of happiness to various levels. Furthermore, not all people prioritize happiness over other components of subjective well-being. We argue that this cultural variation is both widespread and systematic. To lend empirical support to our reasoning, we reanalyze data on ideal levels of personal life satisfaction—the most common operationalization of happiness in psychology—from 61 countries, and we also consider cultural and ecological factors as possible drivers of this variability.

## Happiness and Subjective Well-Being

### Subjective well-being, happiness, and life satisfaction in most prior work (narrow model)

#### Subjective well-being

Researchers tend to define subjective well-being as a construct that refers to subjective evaluations of one’s quality of life (Armenta et al., 2015; Diener et al., 1995). The adjective “subjective” distinguishes the psychological essence of well-being from favorable circumstances (Raibley, 2012) and from objective qualities of one’s life (e.g., physical health). Thus, subjective well-being carries the potential to accommodate a variety of concepts. Indeed, a large number of variables has been used to predict or operationalize subjective well-being, including happiness, life satisfaction, contentment, positive affect, lack of negative affect, relationship flourishing, belongingness, family life, meaning, harmony, self-autonomy, self-actualization, strong relationships, optimism, achievement, health, leisure, hedonism, eudaimonia, spirituality, and psychological richness (Delle Fave et al., 2016; Fowers et al., 2016; Krys, Zelenski, et al., 2019; Uchida & Oishi, 2016).

**Fig. 1.**
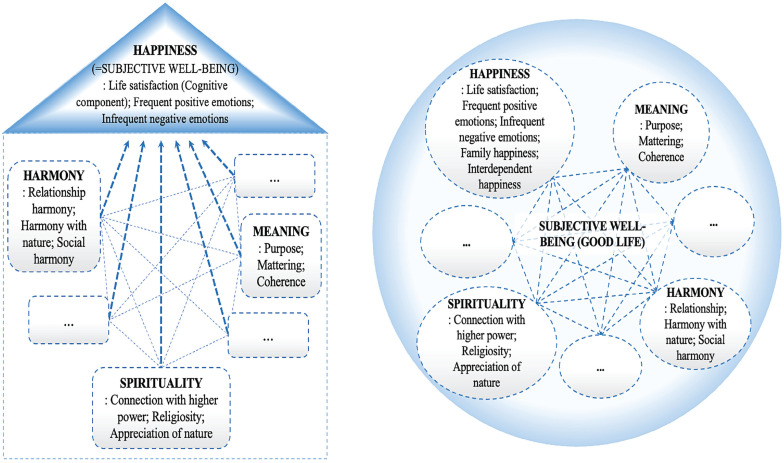
Narrow and broad models of subjective well-being. The narrow model (left) features subjective well-being and happiness synonymously and as superordinate to other components of subjective well-being. The broader model (right) proposes an interdependent network of various components of subjective well-being, with happiness being one of its several components.

Yet these tentative markers have not been treated as equally diagnostic of subjective well-being. The dominant approach to subjective well-being in psychological science, building on Diener (1984, 2000), is to focus on specifically three core facets of subjective well-being: its cognitive evaluation, called life satisfaction; the frequent experience of positive emotions; and the infrequent experience of negative emotions. This model—and in particular the facet of life satisfaction—has arguably become hegemonic in subjective well-being research. Accordingly, other important markers of subjective well-being are frequently treated as feeding into this overarching set of three variables rather than being pursued as end states in their own right (Fig. 1, left).

#### Happiness

Over time, much of psychological science has treated subjective well-being as equivalent to happiness. Indeed, recent definitions of these two concepts are very close—perhaps effectively indistinguishable. For instance, the *Encyclopedia of Quality of Life and Well-Being Research* (Michalos, 2014, p. 6437) states that subjective well-being is “a person’s cognitive and affective evaluations of his or her life” and that happiness is “the degree to which a person evaluates the overall quality of his/her own life as a whole positively”; happiness and subjective well-being essentially became synonyms in past work.

#### Life satisfaction

Although life satisfaction is just one of three independent components of subjective well-being in the model developed by Diener and colleagues, psychological literature nonetheless often uses life satisfaction as a placeholder for happiness and subjective well-being as a whole (Armenta et al., 2015; Diener et al., 1995, 2018). Indeed, life satisfaction is cross-defined with the definitions of happiness and subjective well-being mentioned above, with the *Encyclopedia of Quality of Life and Well-Being Research* (Michalos, 2014, p. 5654) stating that it “refers to subjective well-being and constitutes a cognitive, overall judgement.” The same encyclopedia defines subjective well-being as “a person’s cognitive and affective evaluations of his or her life.” Life satisfaction has become such a popular placeholder for subjective well-being and happiness that it is a core index for policymakers (Fabian & Pykett, 2022). For example, the Organisation for Economic Co-operation and Development (OECD) reports that as of 2018, many countries use national accounts of well-being (NAWB; Diener, 2000; Diener et al., 2015), of which the majority operationalize subjective well-being with a life-satisfaction measure (Durand, 2018).

The way Diener and colleagues’ model of subjective well-being is often applied in psychological research can be considered a narrow model of subjective well-being (Fig. 1, left), in which putative sources of subjective well-being—such as meaning, harmony, and spirituality—contribute to it through shaping positive affect, negative affect, and, especially, life satisfaction. Thus, factors such as meaning, harmony, and spirituality contribute to subjective well-being only to the extent that they elevate one or more of its three components that are frequently interpreted as different forms of personal happiness. So, for example, a meaningful life, in the narrow model, cannot reflect high subjective well-being if that life is simultaneously mediocre in happiness.

### Narrow versus broad models of subjective well-being

The narrow model of subjective well-being, in which happiness effectively substitutes the superordinate subjective well-being construct to which subordinate variables contribute, carries an important theoretical implication: It casts happiness as the “ultimate dependent variable” with other factors (e.g., meaning, harmony, spirituality) as contributors to this overarching outcome. For example, the narrow model of subjective well-being predicts that a person would engage in, say, virtuous action because it contributes to happiness and not because being virtuous is itself considered the primary outcome sought. Accordingly, the narrow model suggests that people will prioritize their levels of happiness both in absolute terms and relative to any of its specific subordinate facets. What is more, the foregrounding of happiness as the ultimate criterion of subjective well-being especially reflects individualistic worldviews (Christopher & Hickinbottom, 2008; Joshanloo et al., 2021) and independent self-construals (Markus & Kitayama, 2010; Zhu et al., 2007). One could be tempted to regard this issue as minor by arguing that any subjective well-being conception can ultimately be measured by the extent to which it leads to an individual’s happiness and positive affect. This, however, appears erroneous given the reduced value placed on positive affect by various cultural norms (Tsai, 2007; Tsai et al., 2006), the non-WEIRD definitions of the self as interdependent (Markus & Kitayama, 2010), or the Buddhist non-self-notion that emphasizes that an “unquenchable” pursuit of happiness may prove unattainable or even harmful because it leads to neglecting others, thus harming both the others and the self (Gowans, 2016; Olendzki, 2005).

The popularity of Diener’s (1984) narrow approach to subjective well-being does not imply that there is no room for alternatives. Indeed, as Diener et al. (2018) clarified, “scientists in the field of subjective well-being do not say that the other approaches to a good life are incorrect” (p. 253). If so, what alternative approaches to understanding subjective well-being may prove worthwhile? One attractive alternative is to consider subjective well-being as a network of interdependent concepts (Fig. 1, right), with each of its constituents varying in their relative importance person to person and society to society. In such a model, happiness would be one of many components of subjective well-being rather than its synonym. The attractiveness of such a network model of subjective well-being lies in its potential to accommodate individual and cultural variations in what, at any given moment, is more central to one’s subjective well-being. For some people and cultures, happiness may be central (Diener et al., 2018), whereas for others meaning might be equally or more important than happiness (Baumeister et al., 2013; Ryan & Deci, 2001); some might prioritize factors such as autonomy, environmental mastery, personal growth, positive relations with others, purpose in life, and self-acceptance (Ryff & Keyes, 1995), and yet others may emphasize harmony (Kjell et al., 2016; Kwan et al., 1997), spirituality (Delaney, 2005), or family and close relationships (Delle Fave et al., 2016).

Our proposal to define subjective well-being more broadly resonates with propositions made previously about the significance of other-than-happiness components in the conceptualization and measurement of subjective well-being (Joshanloo et al., 2021; Joshanloo & Weijers, 2014; Lu, 2008; Lyubomirsky, 2007; Raibley, 2012; Ruggeri et al., 2020; Ryan et al., 2008; Ryff & Keyes, 1995). We join these previous voices by echoing their call for using a broader understanding of subjective well-being and propose a further elaboration of Diener et al.’s (1995) model. Furthermore, we present new empirical evidence to support the reasoning that happiness maximization seems a WEIRD-society-specific legacy in research and policymaking. By doing so we aspire to move the subjective well-being research a step further toward accommodating a more culturally sensitive perspective (Akaliyski, 2023; Krys, Dominguez-Espinosa, & Uchida, 2023; Krys et al., 2020; Thomas & Markus, 2023).

Whether to approach subjective well-being narrowly—by equating it to happiness—or broadly—by treating happiness as one of many tentative components—is not merely a matter of definition; it is an assumption that produces different empirical predictions. If subjective well-being is a network of interdependent components, then various “ideal mixtures” of these components are plausible. Some people may idealize happiness above all, others may idealize sense of meaning over happiness, and yet others may idealize spirituality over happiness. We contend that, in recent years, evidence has accumulated, especially in cross-cultural and ecological psychology, that a broader network model of subjective well-being is more suitable than the comparatively narrow alternative. Furthermore, this evidence suggests that cross-cultural departures from the narrow model of subjective well-being are not lone exceptions to an otherwise complete and accurate model but rather represent substantial deviations that are widespread. In the ensuing sections, we review this work.

### Positioning subjective well-being, happiness, and other factors in the current work (broad model)

With the current article we seek to make an incremental step toward fashioning a more culturally sensitive perspective (Krys et al., 2020) in subjective well-being research, arguing for the broad model of subjective well-being. We understand subjective well-being as a person’s sense of living a good life, happiness as a sense of satisfaction, meaning as a sense of existential mattering, harmony as a sense of balance, and spirituality as a sense of connection with the Greater Power (however one defines it—as God, Energy, Evolution, Nature, or any other way). Because it has been quite common to use concepts of subjective well-being and happiness inconsistently—with theory and empirical practice diverging on occasion (e.g., by theoretically identifying life satisfaction as one component of subjective well-being but empirically using life satisfaction as the sole operationalization of subjective well-being)—and to avoid ambiguity as much as possible, we pay special efforts to clarify our understanding of subjective well-being and happiness.

We make the following observations for our broad model of subjective well-being:

1. We hypothesize that people are guided by different conceptions of what kind of life is worth living; life guided by happiness maximization is only one of such conceptions.
2. We use subjective well-being as an overarching concept covering various phenomena (e.g., happiness, meaning, harmony, spirituality) that we propose as putative components of subjective well-being.
3. The various components constituting subjective well-being are expected to be interdependent—in many contexts, change in one component can and will involve change in similar direction (but not necessarily of similar strength) in other components.
4. The various components constituting subjective well-being are conceptually and empirically distinct. Specifically, although related, the components that constitute subjective well-being operate with partial independence, so a high score on one component does not necessitate a high score on another. For example, exhausting prosocial activities may boost one’s sense of meaning but may decrease one’s own happiness (Myslinska-Szarek et al., 2022); at the cultural level, one may find that citizens of happier nations report a lower sense of meaning (see Oishi & Diener, 2014, Table 1).
5. It is possible that the “recipe” for subjective well-being differs across people, cultures, and historical periods. There can be substantial and meaningful variation in ideal levels of each component constituting subjective well-being. Although it seems reasonable that people will likely strive for some positive level of all the subjective well-being components, the pursuit of the highest levels for each need not be a universal goal (Hornsey et al., 2018), and satiation points for various components constituting subjective well-being may differ between cultural clusters (Jebb et al., 2018).^1^

**Table 1.** WEIRD Correlates of Ideal Level of Personal Life Satisfaction

	Western (W)		Educated (E)		Industrialized (I)	Rich (R)	Democratic (D)	WEIRDness metafactor^a^
	Individualism—Hofstede (Hofstede, 2011)	Individualism—Minkov (Minkov et al., 2017)	Expected years of schooling (UNDP, 2017)	Mean years of schooling (UNDP, 2017)	Technological advancement (Welzel, 2013)	GDP per capita (log-transformed; World Bank, 2017)	Democracy Index (Economist Intelligence Unit, 2020)	
Diener et al. (2000) data set								
Zero-order correlation	.43*	.37^+^	.57***	.47**	.59***	.49**	.63***	.56***
Partial correlation	.39*	.31	.54***	.49**	.55***	.43**	.48**	.50***
*N* (number of countries)	31	29	38	38	38	41	38	41
Krys et al. data set								
Zero-order correlation	.30^+^	.61***	.35*	.41**	.40**	.36*	.31*	.46***
Partial correlation	.40**	.52**	.37*	.55***	.48***	.32*	.15	.43**
*N* (number of countries)	42	34	48	48	47	49	48	49
Both data sets combined								
Zero-order correlation	.35*	.51***	.53***	.50***	.56***	.50***	.51***	.56***
Partial correlation	.44*	.45**	.51***	.56***	.56***	.41***	.33*	.51***
*N* (number of countries)	51	44	63	63	62	66	63	66

a Note: For the WEIRDness metafactor, we standardized scores within each of the seven data sets on WEIRDness and calculated the mean for doubled indicators (i.e., for W and for E) from available standardized scores; next, for each country, we calculated the mean of five indicators (W_mean_, E_mean_, I, R, D) from available standardized scores. Partial correlations were used to control for actual life satisfaction; the number of analyzed countries varies depending on the availability of WEIRD indicators. All variance inflation factors < 1.34. UNDP = United Nations Development Programme.

+ *p* < .10. *p < .05. **p < .01. ***p < .001.

For happiness, we make the following additional clarifications:

1. We consider happiness one of several components constituting subjective well-being. It may or may not be the ultimate, sole, or most important in our lives; its position may vary from individual to individual, across contexts, across times, and across cultures.
2. Happiness may have various facets; in the broad model, life satisfaction, positive affect, and infrequent/low negative affect are considered facets of happiness (empirical research commonly treats life satisfaction as happiness, so in the broad model we cohere theorizing with empirical research); according to the broad model the currently understudied concepts of happiness that are less typical for WEIRD cultures (e.g., interdependent happiness, family happiness) are also facets of happiness, however.
3. Although various facets of happiness can be theorized as largely overlapping with each other, the broad model does not determine the specific relations between them—these relations can differ between individuals, cultures, contexts, and times and remain to be studied empirically.

In the empirical analyses presented in this article we focus on the most popular facet of happiness—life satisfaction. There are two reasons behind this approach. The first reason is that up until now, the field has zeroed in predominantly on life satisfaction as a facet of happiness, with other facets of happiness being studied much less frequently. Accordingly, most insights into happiness, and most policy recommendations based on happiness research, come from research on life satisfaction. Testing our reasoning for the most popular facet of happiness therefore corresponds with the largest body of the currently available literature on happiness. The second reason was pragmatic: At the time of running the empirical analyses presented here, two independent large-scale data sets were available that featured idealization of life satisfaction, but we were not aware of comparable two (or more) large-scale data sets with the other facets of happiness. Importantly, one of the two data sets we use in our analyses came from Diener et al. (2000).

Please note that perspectives other than ours on subjective well-being are possible and may well be justified—as Diener et al. (2018) noted as well. We see ours as helpful in shedding new light on WEIRD happiness maximization and in resultant advocating for the broad model of subjective well-being in social sciences; we do not intend to claim that our approach is the only one possible.

## The Conceptualization of Subjective Well-Being Is Varied

Our recent study of 13,000 people across 49 countries investigated what people considered to be their ideal level of happiness by asking about their ideal level of life satisfaction. Of these individuals, 97% indicated that their ideal level of happiness was to be at least “a little happy” or greater (Krys et al., 2023; Krys, Park, et al., 2021; Krys, Yeung, et al., 2022). Participants also indicated whether they wished to be happier than they currently were; as many as approximately 25% of the same participants indicated that they did not despite their actual levels of happiness being below the maximum possible. People want to be generally happy, but for many, only up to a certain point. In fact, only around 15% of these participants indicated that their ideal level of happiness was the maximum possible. These findings are consistent with research showing that people can feel anxious (Joshanloo & Weijers, 2014) or uncomfortable (Miyamoto et al., 2010) about being very happy.

Why do such differences exist, and what factors might explain this variability in attitudes toward happiness? One possibility is that the relative positioning and prominence of happiness, and other components of subjective well-being, may vary systematically across groups. In fact, the central positioning that happiness seems to occupy in conceptualizations of subjective well-being may itself reflect a cultural idiosyncrasy, one especially endorsed within WEIRD societies.

### Subjective well-being in WEIRD societies

From a historical point of view, the foundation of the Western study of happiness derives from Aristotle’s notion of *eudaimonia* (Kesebir & Diener, 2008; Kraut, 2015). Eudaimonia is often translated as happiness and was described by Aristotle as the ultimate goal of human life, a goal that cannot be attributed to any other superordinate goal and toward which all other strivings are instrumental. There are important differences between Aristotle’s eudaimonia and contemporary accounts of happiness—Aristotle considered virtuous activity a particularly important facet (Kraut, 2015), and the Greek term suggests a role for the divine (*eu* = “well”; *daimon* = “divinity” or “spirit”). Nonetheless, contemporarily definitions of happiness are more centered around its hedonic aspects.

The topic of happiness featured prominently in the philosophy of the Enlightenment. In *Utilitarianism*, John Stuart Mill argued in work currently considered the philosophical articulation of liberal humanism that “we are morally obliged to follow those social rules and precepts the observance of which promote happiness in the greatest extent possible” (Mill, 2014, p. 17). In its original form, utilitarianism proposes that pleasure is a quantifiable positive result for the greatest possible number of people and constitutes the criterion for making decisions. This consideration of happiness, which accompanied the technological and intellectual developments of the Enlightenment, even left its mark on the American Declaration of Independence. Therein, the right to pursue happiness is explicitly established, and the idea that happiness should be actively sought and promoted gained traction in much of modern psychological science (McMahon, 2008).

European and North American contemporary understandings of happiness are related to “feeling well,” which being primarily a subjective issue, lies within the control of the individual (Kesebir & Diener, 2008). Oishi et al. (2013) proposed that these understandings of happiness emanate from the economic history of WEIRD societies, the rise in consumer culture within these societies, and the increased use of individual emotions in advertisement and discourse. Psychological studies consistently feature happiness—operationalized most often using measures of life satisfaction—prominently in work on subjective well-being and elsewhere, as noted by Kwan et al. (1997):

An ultimate dream for everyone in the field of psychology is to understand human behaviors, so that psychology can contribute to people’s well-being. On the basis of this common goal, a single construct of life satisfaction, which illustrates “the highest good” and “the ultimate motivator” for all human behaviors, has drawn continuous attention for the past few decades. (p. 1038)

Studies on happiness—operationalized as life satisfaction—are possibly among the most popular in psychology. According to the Scopus database, as of January 2023 Diener and colleagues’ (1985) article introducing the Satisfaction with Life Scale (SWLS) used to quantify subjective well-being has been cited over 16,000 times, making it one of the most impactful articles in the psychological literature (cf. Ho & Hartley, 2016).

### Subjective well-being in non-WEIRD societies

An examination of non-Western philosophical traditions reveals that the centrality of happiness for subjective well-being may be more tenuous than commonly assumed in the psychological literature. For example, Buddhist teachings from Eastern Asia emphasize that the source of suffering is located in thirst or craving and that an unquenchable pursuit of happiness may prove unattainable (Gowans, 2016) or even harmful because it leads to neglecting others, thus harming both the others and the self (Joshanloo & Weijers, 2014). To derive high subjective well-being in the Buddhist view, people must instead free themselves from wants and desires (Sundararajan, 2008). Thus, the Buddhist state of “desirelessness” stands in contradiction to the European emphasis on satisfaction, a term that originated from the Latin *satisfacere* (*satis* = enough; *facere* = make) and literally meaning “meeting the expectations, needs and desires” (Oxford English Dictionary, n.d.). What in European philosophy represents the ultimate life goal can from a Buddhist perspective be characterized as a pathway to an unfulfilled and frustrating life.

A similar contradiction of ideals about subjective well-being can be found in Japanese culture. Miyamoto et al. (2010) showed that in the Japanese cultural context the experience of happiness can be accompanied by a fear of troubling others. In Japan, the individual experience of happiness carries significant ambivalence unknown in WEIRD societies. Furthermore, being satisfied may entail a conflict with the important Japanese value of constant self-improvement. Probably one of the most common sayings in Japan is 頑張って (*ganbatte*), which means “do your best.” Japanese culture emphasizes the value of interpersonal harmony, ordinariness, and quiescence, which are regarded as components of interdependent happiness (Hitokoto & Uchida, 2015).

Joshanloo (2013) compared Western versus Islamic traditions and found that the Islamic conception of subjective well-being is more related to striving to do the right thing and is closely related to religiousness. Muslim theology endorses submission to the divine will and encourages an ascetic way of life for the soul to be kept aligned to “the right way.” According to the Islamic worldview, human nature has an innate tendency to forget the right path and wander if not continuously reminded. Succumbing to happiness bears the risk of distracting people from following the right way. Similar to Islam, among various interpretations of the Talmud (i.e., in Judaism), subjective well-being is related to following the precepts of God (Levi, 2014).

In another study, Joshanloo (2014) analyzed differences between Eastern (i.e., Buddhist, Hindu, Taoist, Confucian, and Sufi) and Western concepts of subjective well-being and described six major contrasts between these two cultural groupings. Subjective well-being is associated with self-transcendence or self-enhancement, eudemonism or hedonism, harmony or mastery, contentment or satisfaction, valuing or avoiding suffering, and relevance or irrelevance of spirituality and religion. Joshanloo acknowledged that both Eastern and Western philosophical traditions recognize all 12 tendencies but noted that their relative importance varies. Happiness—as is commonly studied by psychological science—is closer to life satisfaction, hedonism (e.g., positive affect), and avoiding suffering (e.g., absence of negative affect), which in Joshanloo’s classification are priorities characteristic of Western societies.

Some African conceptualizations of subjective well-being stress the importance of harmony. Harmony can be understood as the result of directing human action to achieve a transcendence that is derived from being in synchrony with the physical and the social world (Asante, 1984). According to several strands of African thought, directing one’s action toward harmony with the social world (Mandela’s “obuntu”) is necessary to achieve a state of godly “possession”—perceiving oneself to be living and acting in accordance with the will of the gods. It also involves a cleansing of one’s spirit, a state that is believed conductive to euphoria, entailing a sense of peace with others (Asante, 1984).

Furthermore, Joshanloo and Weijers (2014) proposed that some individuals, mainly from non-WEIRD cultures, are averse to happiness. They identified four beliefs that underlie an aversion to happiness: First, achieving happiness makes it more likely that bad things will happen; second, pursuing happiness causes a happy person to become a bad person; third, attaining happiness is bad for the happy person and others; and fourth, pursuing happiness is bad for others. However alien these four beliefs may seem to many from WEIRD cultural backgrounds, the existence of aversion to happiness in non-WEIRD cultures lends another line of support to our argument that, in non-WEIRD cultural traditions, happiness is not the key component of subjective well-being and may in some cases or at some levels even be considered detrimental to it.

### Happiness does not rank among the most important values

Value-taxonomy research from Schwartz (2009) offers further insights about the putative central position of happiness in the context of subjective well-being. Although the 57 values studied by Schwartz did not refer to happiness per se, certain values analyzed by Schwartz (e.g., “enjoying life” or “pleasure”) overlap with happiness, whereas other values (e.g., “meaning in life” or “inner harmony”) overlap with other types of subjective well-being. Importantly, in Schwartz’s mapping, these happiness-related values are located on the opposite side of his value circumplex from those values related to other types of subjective well-being. From this perspective, happiness-related values, at least to the extent that they reflect enjoying life and pleasure, constitute only a part of the universal-values circumplex, and other types of subjective well-being are valued as different from, if not opposite to, happiness.

Of Schwartz’s seven cultural orientations, “egalitarianism” received the highest endorsement. “Affective autonomy,” which reflects a happiness-related value, was the second least endorsed cultural orientation. Importantly, endorsement for Schwartz’s “affective autonomy” is correlated with cultural individualism (Inglehart & Oyserman, 2004; Krys, Uchida, et al., 2019). Thus, Schwartz’s mapping of cultural values lends further support for questioning that happiness is the ultimate aim and the highest type of subjective well-being universally and may in fact be a distinctive feature of WEIRD societies.

## Ideal Levels of Happiness Vary Systematically Along WEIRD Cultural Factors

As discussed above, there is considerable variation in the degree to which people strive for maximum happiness, and these differences seem in part rooted in various sociocultural sources, such as worldviews and religions, languages, and cultural beliefs. Furthermore, people’s ideal levels of happiness vary systematically along WEIRD cultural factors.

We examined two large cross-cultural data sets in which people reported their ideal levels of personal life satisfaction (Diener et al., 1985)—the most common operationalization of happiness in psychology—and correlated these ideal levels of happiness with macrolevel indicators of WEIRD cultural factors. The data we used were from Diener et al. (2000; 7,167 participants across 41 countries) and from Krys and collaborators (Krys et al., 2023; Krys, Park, et al., 2021; Krys, Yeung, et al., 2022; 12,819 participants across 49 countries).

For both data sets, participants rated their actual and ideal level of happiness using the SWLS (Diener et al., 1985). Specifically, we, and Diener before us, asked how participants thought the ideal person would complete SWLS items. The instruction for the ideal levels of happiness ratings, using SWLS from (Krys et al., 2023; Krys, Park, et al., 2021; Krys, Yeung, et al., 2022) read as follows: “Instead of answering how much you agree with the statements, we would like you to indicate how much you think the ideal or perfect person would agree with each statement.” Participants answered these questions about their actual and ideal level of happiness on the SWLS using a 9-point scale (1 = *doesn’t describe him/her at all*, 3 = *describes him/her a little*, 5 = *describes him/her moderately*, 7 = *describes him/her very well*, 9 = *describes him/her exactly*) per Vignoles et al. (2016). The Diener et al. (2000) data set featured the same measures of actual and ideal happiness levels, but the responses ranged from 1 (*strongly disagree*) to 7 (*strongly agree*). We present analyses for both data sets separately but also for their combination (i.e., we standardized scores within each data set, and then for each country we calculated means from these standardized scores).

We obtained WEIRDness indicators at the macro (country) level. Accordingly, we operationalized Westernization using two indexes of individualism (Hofstede, 2011; Minkov et al., 2017). We used the expected and mean duration of schooling in a country from the corresponding United Nations Development Programme (2017) index as an indicator for education. Further, we operationalized industrialization through the technological-advancement index established by Welzel (2013). GDP per capita, estimated by the World Bank (2017), served in our analysis as the marker of richness. Finally, as a measure of the level of democracy we used *Democracy Index 2019* (Economist Intelligence Unit, 2020).

### WEIRD societies set higher happiness ideals

Our two data sets showed that countries that feature high happiness ideals also feature relatively higher average citizen happiness (*r*_Diener et al._ = .68, *r*_Krys et al._ = .83, *p*s < .001). Given their correlation, we controlled for actual happiness when analyzing the associations between ideal happiness and WEIRD factors. We summarize these partial correlations in Table 1. Strikingly, in both data sets, six of the seven indicators of WEIRDness—individualism (2x), education (2x), technological advancement, GDP per capita, and level of democracy—were positively and significantly partially correlated with ideal happiness (level of significance was not reached by Minkov’s individualism analyzed with the Diener data set or for the democracy index analyzed with the Krys et al. data set).

When we combined both data sets, we found no exceptions. Thus, the stronger a country was characterized by WEIRD factors, the more its inhabitants subscribed to high happiness ideals independent of their actual levels of happiness. These data provide a compelling case that the relatively strong idealization of happiness is specific to WEIRD societies. Accordingly, assuming the pursuit of happiness to be at the center of subjective well-being, superordinate to other facets of subjective well-being, may not be as uncontroversial as has been assumed in subjective well-being research.

Both Northwestern European and Latin American societies tend to occupy top ranks in actual happiness rankings (e.g., Minkov, 2009; Veenhoven, 2016). However, whereas Northwestern European societies tend to be WEIRD, Latin American societies tend not to be. In the Supplemental Material available online, we document that Latin America and Northwestern Europe scored comparatively high on average actual happiness but that at the same time the average ideal happiness was significantly lower in Latin American countries than in Northwestern European countries. These findings further emphasize our theorizing that the idealization of high happiness is more cross-culturally variable than typically theorized and that the ideal levels of happiness depend on a country’s WEIRD status.

Cultures differ in the extent to which individuals are willing to select extreme responses. With happiness scores tending to fall above midpoint, country differences in extreme responses could bias scores toward higher levels of average happiness. Studies show that WEIRD cultures are characterized by low bias, with extreme responses more prevalent in the less developed countries (Meisenberg & Williams, 2008; Smith, 2004). In the empirical analyses we present in this article, Northwestern Europe is at the top of the “happiness-maximization” rankings, and the regions characterized as gravitating toward high extremity in responses occupy bottom to middle positions (see the Supplemental Material). Therefore, we found support for our predictions *despite* any potential impact of extremity in response style.

## Possible Drivers of Happiness Idealization in WEIRD Societies: Ecology, Geography, and Economy

Previous sections emphasized that specific worldviews, religions, ideologies, languages, and sociohistorical contexts affect how happiness and subjective well-being are conceptualized and furthermore showed that ideal levels of happiness—operationalized as life satisfaction—vary systematically along factors characterizing WEIRD societies. Why do such systematic differences, and especially those between WEIRD and non-WEIRD societies, exist? A growing body of research links cultural differences, including the psychology of happiness, to ecological and socioeconomical conditions (Kim, 1995; Welzel, 2013). Specifically, the proliferation of happiness as central and as a subordinate feature of subjective well-being in WEIRD (vs. non-WEIRD) societies may be cultivated by these societies’ historical interaction with their ecological environment.

### Ideal happiness varies by existential pressures

Previous studies have linked ecological pressures to societal subjective well-being. Specifically, low existential pressures (manifested as a climatic configuration called the cool-water condition^2^ and high pathogen security) have been found to predict high levels of actual happiness (Welzel, 2013; Welzel et al., 2021). Previous research has also indicated that a sense of freedom—comparatively common in WEIRD societies—is a mediator between existential pressures and happiness (Welzel, 2013). Liberating people from existential pressures enhances opportunities for pursuing happiness and focusing governing bodies on promoting citizen happiness (Welzel, 2013).

Thus, low existential pressures may lead to higher levels of happiness idealization. If so, then showing this relationship would provide further support for the notion that the centrality and prominence of happiness as a superordinate outcome in models of subjective well-being is questionable.

We propose that societies may be more likely to develop a happiness-maximization principle if set in comparatively benign ecological conditions, or as Welzel (2013) framed it, in cultures that faced the smallest existential pressures. Societies inhabiting the most convenient ecological habitats can allocate their resources—time, workforce capacities, materials—not only for everyday survival (i.e., “escape from suffering”) but also for *joie de vivre*. This is, they can afford to idealize happiness maximization.

We utilized the cross-cultural data on actual and ideal happiness gathered by Diener and colleagues (2000) and those collected by Krys et al. (2023), Krys, Park, et al. (2021), and Krys, Yeung, et al. (2022) to examine whether ideal levels of happiness might systematically vary across ecological indicators of existential pressure. We operationalized these ecological factors as (a) the cool-water index (Welzel, 2013; after Gallup et al., 2010), (b) historical pathogen prevalence (Welzel, 2013; after Murray & Schaller, 2012), and (c) the risk of natural disasters (World Risk Report, 2019). As in our previous analyses, we correlated these three markers of benign ecology (cool-water index, pathogen safety, and natural-disaster security) with ideal levels of happiness (see Table 2) with and without controlling for differences in actual happiness.

**Table 2.** Correlations Between Existential Comfort (Convenience of Ecological Habitats) and Ideal Level of Personal Life Satisfaction

	Cool-water index (Welzel, 2013)	Disease security (Welzel, 2013)	Natural-disaster security (World Risk Report, 2019)	Existential-comfort metafactor
Diener et al. (2000) data set				
Zero-order correlation	.52***	.40*	.48**	.52***
Partial correlation	.48**	.21	.40*	.40*
*N* (number of countries)	38	38	36	38
Krys et al. data set				
Zero-order correlation	.41**	.54**	.26+	.49***
Partial correlation	.48***	.58***	.35*	.57***
*N* (number of countries)	48	46	46	48
Both data sets combined				
Zero-order correlation	.53***	.49***	.28*	.53***
Partial correlation	.53***	.41**	.34**	.52***
*N* (number of countries)	63	61	60	63

Note: For the existential-comfort metafactor, we standardized scores within each of the three data sets on existential comforts, and then for each country we calculated the mean from available standardized scores. Partial correlations were used to control for actual life satisfaction; the number of analyzed countries varies depending on the availability of WEIRD indicators. All variance inflation factors < 3.23. WEIRD = Westernized, educated, industrialized, rich, and democratic.

**Fig. 2.**
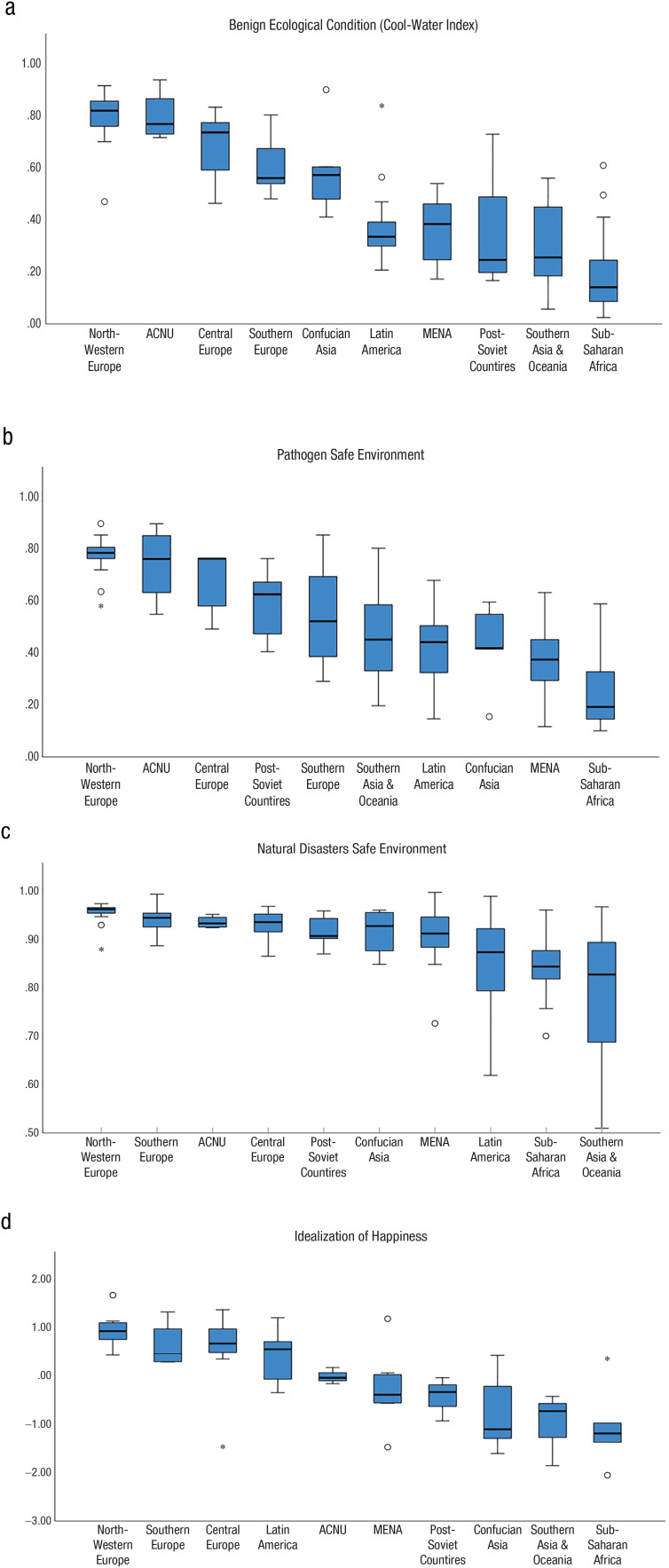
Northwestern European eco-environmental habitats are benign compared with those of other regions. Box plots for (a) the cool-water index (convenient temperature, rainfall continuity, and the abundance of ice-free waterways), (b) pathogen safety, (c) natural-disaster security, and (d) idealization of happiness are arranged in descending order of mean scores. Lines in the middle indicate the median level, boxes indicate the 25th and 75th percentiles, whiskers indicate the minimum and maximum values of the data within the range extending 1.5 times the height of the box, and circles and asterisks are outliers (values that do not fall within whiskers; asterisks are from the box at least three times away from the height of the box). Northwestern Europeans idealize happiness most (despite being low on extreme responding). ACNU = Australia, Canada, New Zealand, and the United States; MENA = Middle East and North Africa.

The results of these analyses confirmed that ideal levels of happiness vary systematically along levels of the ecological factors, with the presence of cool and navigable waters, security from diseases, and absence of natural disasters each predicting higher ideal levels of happiness, even after controlling for actual happiness (with the exception of disease security not strongly predicting ideal happiness in the Diener et al. data set with actual happiness controlled, *p* = .20). This set of findings indicates that how strongly societies idealize high levels of happiness relates to the existential threats arising from the ecology they occupy (see also Fig. 2).

**Fig. 3.**
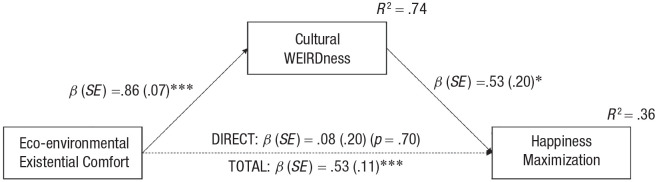
The cultural syndrome of WEIRDness mediates the association between benign eco-environmental conditions and happiness maximization. WEIRD = Western, educated, industrialized, rich, and democratic.

To further evaluate our reasoning that exceptional eco-environmental conditions might have fostered the emergence of the cultural syndrome of WEIRDness, which in turn led to the emergence of happiness maximization, we ran a mediation analysis using jamovi open-source software (Version 1.6.23.0; medmod module; 5,000 bootstrap samples) in which existential comfort (see Table 2) served as the independent variable, cultural WEIRDness (see Table 1) as the mediator, and ideal level of life satisfaction as the dependent variable. A significant indirect effect supported our reasoning, *IE* = .45, *SE* = .19, 95% CI = [.09, .86] (see Fig. 3).

### The role of the Gulf Stream in shaping the Northwestern European habitat

**Fig. 4.**
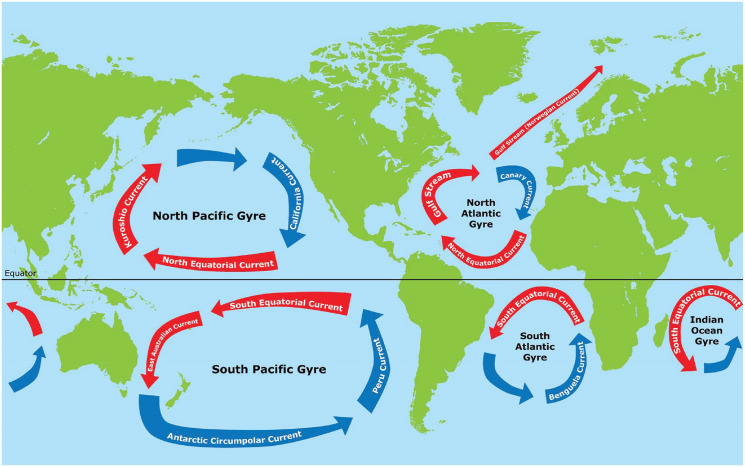
Global flow of currents. The Gulf Stream (Norwegian Current) is an exception on a planetary scale and makes the European habitat exceptionally benign for human habitation.

Europe lies in the geographical latitude of the mild climate zone. Additionally, the climate of Northwestern Europe is milder than in other regions of similar geographical latitude because of the North Atlantic drift of the Gulf Stream (Palter, 2015; see Fig. 4). The Gulf Stream is an exceptional phenomenon on a planetary scale (see Fig. 4; see also Latif et al., 2021). Surface oceanic waters provide a regular pattern of currents, but the Gulf Stream breaks out from this scheme, making European climate exceptionally agreeable for human life.

The general pattern of global oceanic currents works as follows: Along the equator, surface-level oceanic waters flow from east to west, absorbing heat and humidity from the skies above. Next, when deflected by the eastern coasts of continents, currents turn northward in the Northern Hemisphere and southward in the Southern Hemisphere, providing heat and increasing humidity along the eastern coasts of adjacent continents. After reaching the latitude of the tropics (Tropic of Capricorn in the Southern Hemisphere and Tropic of Cancer in the Northern Hemisphere), currents start flowing from west to east. When currents reach the western coasts of the continents, they are already carrying cold waters with dry air above. Finally, currents turn along western continental coasts back toward the equator. The whole process is one of constant, circular dynamics (see Fig. 4). This process has important consequences for climatic conditions of mid-latitude eastern and western coasts of continents. Whereas the climate of the eastern coasts at the mid-latitudes tends to be humid and tropical, the climate of the western coasts at the mid-latitudes tends to be dry and desertic. The most striking examples of this process are Florida versus the California peninsula, East Asia versus the Sahara, Amazonia versus the Atacama Desert, Madagascar versus the Namibian Desert, and the eastern versus western coasts of Australia.

The only exception from the general pattern described above is the Gulf Stream that carries warm water and humid air northward along the northwestern coast of the European continent. The Gulf Stream warms and humidifies the Northwestern European climate (in particular during winters that would otherwise be colder and dryer). Because of this exceptional oceanic current, Northwestern European winters are up to 10 °C warmer than the zonal mean at equivalent latitudes (Palter, 2015). To illustrate this difference: Paris is 5° geographical latitude closer to the North Pole than Vladivostok (48.86°N vs. 43.12°N), and so Paris should be several degrees colder. Yet the temperature in the coldest month is around +3 °C in Paris and around −18 °C in Vladivostok. The Gulf Stream also provides Europe with humidity—without humid air brought by the Gulf Stream, Europe would be desertic and drier, particularly in winters.

In effect, because of exceptionally mild winters, the period for vegetable growing in Western Europe is much longer than in other regions of similar latitude. At the same time, the temperature in Europe remains milder than in Sub-Saharan Africa, Amazonia, or Sumatra. Therefore, in Europe, plagues of disease-provoking germs, bacteria, and insects were historically much rarer. Already before the modern era, the relatively pathogen-safe European environment afforded more safe water, a decreased prevalence of communicable diseases, and reduced child mortality (Welzel et al., 2021), thus fostering the idealization of relatively higher levels of happiness.

In sum, the Northwestern European climate is shaped by the exceptional oceanic current of the Gulf Stream. For ages, it has made Northwestern European eco-environmental conditions easier for human life (i.e., it has lessened existential pressures; Welzel, 2013). Thus, we propose that these exceptional eco-environmental conditions might have fostered the emergence of cultures prioritizing happiness maximization. In Northwestern Europe, it was easier to escape much human suffering and in consequence to idealize high levels of happiness.

## Happiness Maximization as a Cultural Syndrome

The above sections showcase the widespread and systematic variation in the conceptualization of subjective well-being, the importance assigned to happiness, and in the ideal levels of happiness that people pursue. We also consider ecological and economic factors to be possible drivers of this variability. The fact that happiness appears to be idealized in regions with comfortable ecological and economic conditions should not lead to the conclusion that it might be a goal suitable for universal pursuit. Our point is that happiness maximization may have developed as a cultural syndrome but that as such it may entail negative consequences as well. Cultural syndromes express variations of psychological constructs found in particular societies during certain time periods (Triandis, 1996). Following this rationale, if happiness maximization is a cultural syndrome, negative consequences may occur from the core elements of the concept and involve attempts to increase positive feelings and possibly attempts to suppress negative feelings (Oishi et al., 2007, 2014; Yeung & Lun, 2016). To tentatively evaluate this idea, we analyzed the association of happiness idealization with two categories of negative consequences: side effects and opportunity costs.

### Societal correlates of happiness maximization

Societies in which comparatively many people strive for happiness maximization tend to feature a number of indicators that may be seen as problematic, such as the use of psychotropic substances (perhaps because these substances often aim at promoting positive feelings or suppressing negative ones) and heightened levels of mania (i.e., pathologically elevated, mood-enhancing behaviors).^3^

#### Happiness maximization and substance use

In the Supplemental Materials, we present the results of the country-level correlational analyses of association between happiness maximization and the use and abuse of nine drugs based on our data and data from the United Nations Office on Drugs and Crime (2019). It turns out that cultural happiness maximization correlates with the general use and abuse of drugs at the level of country averages (we combined data on several drugs into a metafactor), with the association varying from *r* = .13 to *r* = .37 (depending on the data set, on how broadly we measured the drug metafactor, and whether we controlled for actual levels of happiness); *r* oscillated around .30 for the analyses run on ideal happiness combined for both data sets. Among various substances analyzed, cannabis and tranquilizers—both associated with lessening stress and pressures—seemed to play a special role in societies of happiness maximization (with .25 < *r*s < .43 when both data sets are combined).

The Supplemental Material also presents results of country-level correlational analyses from five various data sets on alcohol use and abuse (World Health Organization, 2019). Happiness maximization at the cultural level was significantly associated with higher average alcohol use and abuse. The corresponding correlation between their metafactors ranged from .27 to .60. When the datasets were combined, ideal happiness and alcohol use and abuse metafactor had correlations of above .40. Living in happiness-maximization cultures means, on average, higher alcohol consumption by members of that culture. When WEIRDness was controlled for in these analyses, the associations between happiness idealization and the substance use faded away. These results suggest that WEIRDness, happiness maximization, and substance use are positively related to each other at the level of country aggregates. Thus, (WEIRD) societies in which people seek to maximize happiness more also tend to be societies in which substance use is higher. Of course, the causal nature of this association, or indeed whether those who seek to maximize happiness are the same individuals to use substances, is not certain.

#### Happiness maximization and bipolar disorder

Scholars have suggested that an increased prevalence of bipolar disorders may have roots in the pathoplastic (shaping) role of culture on mental disorders in general (Haroz et al., 2017; Kirmayer & Ryder, 2016; Koelkebeck et al., 2017; Kuo et al., 1989; Ryder et al., 2008). Happiness maximization may be a part of that process. Bipolar disorder, for example, encompasses manic episodes of highly elevated mood (Carvalho et al., 2020), states that can be associated with pursuing states of maximum happiness. The country-level data confirmed our prediction that high ideal levels of happiness in a society relate positively to the prevalence of bipolar disorder, with .16 < *r*s < .62; for both data sets the combined *r* values are > .40 (see Supplemental Materials). Importantly, when the cultural syndrome of WEIRDness was controlled for, the correlation between idealizations of high levels happiness on bipolar disorder maintained its significance, albeit weaker. Societies whose individuals pursue high levels of happiness thus tend to be societies with a relatively high occurrence of bipolar disorder, although the causal nature and whether these elevated levels occur in the same individuals are again open questions.

#### Beware of committing the ecological fallacy

We call attention to the fact that we analyzed country-level phenomena. Our conclusions cannot be extended to the individual level without the risk of committing the ecological fallacy (e.g., the fact that the country-level association between happiness maximization and substance use is positive does not mean that individuals who seek maximum happiness tend to use substances). We are dealing with societal-level, not individual-level, phenomena, and ecology is different from psychology (Leung & Bond, 2007). We are aware that societal phenomena are shaped by multiple determinants and that happiness maximization represents but one among several variables therein. The reason we point to some perhaps overlooked associations is to raise awareness that happiness maximization as a cultural syndrome appears to be correlated with advantages and disadvantages. This perspective stands in opposition to viewing happiness as the ultimate dependent variable and opens new perspectives for the future study of the related phenomena.

### Opportunity costs of happiness maximization

Happiness maximization may encompass opportunity costs (Arce, 2016; Ferraro & Taylor, 2005; Parkin, 2016; Potter & Sanders, 2012), meaning that pursuing happiness may lead to missing out on the positive aspects of alternative forms of subjective well-being. In fact, Oishi and Diener (2014) found that members of the happiest societies report a lower meaning in life than do members of moderately happy societies (*r* = −.33). To the best of our knowledge, the Oishi and Diener study that used Gallup data (Oishi & Diener, 2014) is the only study that has compared people’s declarations about meaning in their life across a large number of countries. As of 2022, happiness has been studied in several large cross-cultural research projects (e.g., see Diener, 2000; Gallup, 2022; Gardiner et al., 2020; Haerpfer et al., 2020; Helliwell et al., 2022; Krys et al., 2023; Krys, Park, et al., 2021; Krys, Yeung, et al., 2022), whereas meaning in life has been studied in one large cross-cultural scheme (Gallup, 2022; after Oishi & Diener, 2014). Large cross-cultural studies on alternative forms of subjective well-being are also scant. This evidence illustrates the imbalance in attention directed at happiness and at other types of subjective well-being despite their geographical and cultural variation.

In the current section we illustrated the potential side effects of happiness maximization by considering its arguably undesirable society-level correlates. We did so to offer some balance to the otherwise predominant focus on positives associated with happiness (e.g., Inglehart et al., 2008), thus illustrating that happiness maximization may not be uniformly desirable.

## Discussion

It is generally good to be happy. To what extent people idealize happiness, however, is an unobvious but empirically tractable question that remains largely understudied. On the basis of recent work in cross-cultural psychology, we propose that the traditionally central and dominant position that happiness has occupied in models of subjective well-being needs to be reassessed. The conceptualization of subjective well-being and happiness and the beliefs surrounding them differ substantially across societies, with some societies celebrating the pursuit of high levels of happiness, whereas others do not. Data on values and happiness confirm these suggestions: Happiness is not considered a top-ranked value in many societies, and people do not universally wish to maximize it. Rather, it seems that the central positioning of happiness in models of subjective well-being may represent a priority of WEIRD societies in particular and not for other societies across the globe. Given that these cross-cultural differences are systematic and testable, we propose that more differentiated models of subjective well-being should be extended into cross-cultural consideration, and in the current work we presented such a model (see Fig. 1).

**Fig. 5.**
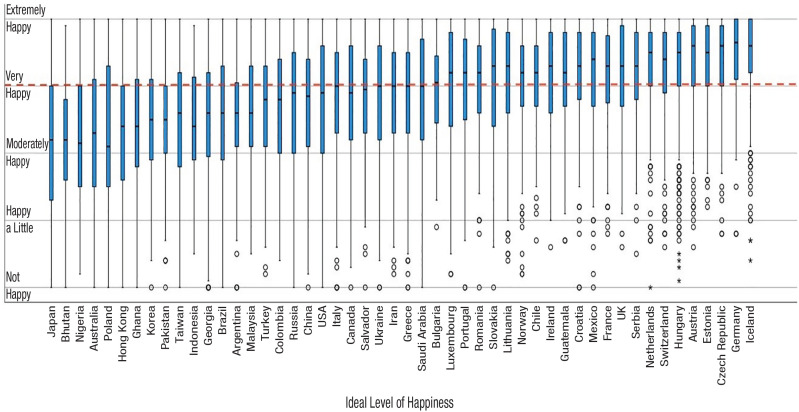
Ideal level of happiness across cultures. Using a more articulated theoretical rationale and the data from 19,986 participants from 66 countries, the current article aims to show that the idealization of high levels of happiness is shaped, among other factors, by cultural context and that the ideal of “happiness maximization” emerged particularly in Northwestern European cultures. We further propose that the idealization of high levels of happiness may have evolved in cultures that emerged in the most benign eco-environmental habitats (i.e., those that have faced the smallest existential pressures). Northwestern Europe has the most benign eco-environmental conditions of all large macrogeographical regions of our planet, and, drawing from geographical science, we indicate the probable role of the Gulf Stream in creating them. The red dashed line illustrates “very happy,” the line in the middle indicates the median level, boxes indicate the 25th and 75th percentiles, whiskers extend to 1.5 times the height of the box, and circles and asterisks are outliers (values that do not fall within whiskers; asterisks are from the box at least three times away the height of the box). Source data are from Krys et al. (2023), Krys, Park, et al. (2021), and Krys, Yeung, et al. (2022).

It is important to underscore the substantial magnitude of the cross-cultural differences in ideal levels of happiness that we have established in our data sets. In our study, the difference between Northwestern Europe and the rest of the world reached Cohen’s *d* = 1.93, *M*_Northwestern Europe_ = 7.45, *SD* = .30; *M*_other countries_ = 6.51, *SD* = .67; *t*[47] = 4.29; *p* < .001. To put this difference more concretely, the Krys et al. data set^4^ showed that in Germany and Iceland 86% and 84% of participants respectively indicated that ideal happiness is “very happy” at least; in Bhutan, Ghana, Nigeria, Japan, and Pakistan, 70% or more participants indicated that ideal happiness is below “very happy” (with Bhutan reaching 81.5%). These are substantial differences, having potentially major consequences for people’s mindsets and their way of being in life. For an illustration of ideal levels of happiness across all cultures studied by (Krys et al., 2023; Krys, Park, et al., 2021; Krys, Yeung, et al., 2022), see Figure 5.

### Implications for psychological science

Our findings are in line with the premise of mutual constitution between culture and psychology, according to which psychological phenomena evolve in interdependence with the social and physical environment in which they appear and, as a result, vary cross-culturally (Markus & Hamedani, 2007). Furthermore, they add to the large body of research on evolutionary psychology that shows that several theoretical assumptions based on WEIRD samples are often too narrow to accommodate subsequent evidence (Arnett, 2008; Henrich et al., 2010; Krys, Vignoles, et al., 2022; Thalmayer et al., 2021). As a way forward in transcending WEIRDness, Barrett (2020) suggested a phenomical approach to psychology. Borrowed from recent evidence in biology, this approach involves mapping several variations of a psychological phenomenon of interest and showing how each variation evolves in relation to the environment. Our findings align with this perspective in conceptualizing happiness as one of many components of subjective well-being, shaped by evolutionary and cultural factors. This idea enlarges our understanding of subjective well-being and bears a series of implications for psychological science and practice.

#### Toward cultural objectivity in subjective well-being studies

Placing happiness within a broader conception of subjective well-being is not only an analytical issue. It also touches on issues of ethics and power because adopting a WEIRD-based conceptualization of happiness may presume a stance of dominance toward other forms of subjective well-being. All cultures deserve to be accommodated in developing and testing theories of subjective well-being. Applying WEIRD theorizing on subjective well-being to other parts of the world bears the risk of imposing WEIRD standards to “educate” or “help develop” others, often people in former colonialized regions. Psychological science has become more and more aware of the neocolonial nature of such presumption (Bulhan, 2015; McNamara & Naepi, 2018; Okazaki et al., 2008; San Juan, 2006). Recognizing that happiness is not tantamount to subjective well-being and that subjective well-being is a much more complex and multidimensional phenomenon is a step toward decolonizing psychology. From a cultural perspective, it is hard to imagine a universal recipe for a good life. It is possible and necessary, however, to study the cultural variety in recipes for living a good life.

#### The need to elaborate a pattern of subjective well-being types

If happiness is not the ultimate dependent variable, then the question of developing a taxonomy for types of subjective well-being appears. For example, keeping the Maslow hierarchy (Maslow, 1943) in mind, can we develop a schema of subjective well-being in which happiness is a “lower level” component of subjective well-being and meaning in life a “higher level” component of subjective well-being? That is, do people satiate their happiness first and next their meaning and other components of subjective well-being (Gorski, 2022)? Adopting a more nuanced understanding of subjective well-being poses further questions about possible relationships between subjective well-being components, their cultural congruence, and the degree to which each of them is pursued across societies varying in ecocultural characteristics (Sng et al., 2017).

#### Toward conceptual pluralism in subjective well-being studies

Our study concerns the relationship of happiness to other subjective well-being components within psychology (see Fig. 1). The question here is how to achieve a conceptual pluralism and develop concrete measures that would emanate from the integration of broadened views on subjective well-being. We call for comprehensive reviews of components of subjective well-being and use the cultural perspective to mention a few candidates: *Sense of meaning* might be recognized and accepted at least equally with happiness across many cultural and subcultural groups; a key aspect of good life in Confucian Asia is a *sense of harmony* (e.g., Kjell et al., 2016; Lun, 2022); and it seems difficult to discuss good life in Africa, the Middle East, or Latin America without studying the role of *spirituality*/*religiosity* (Saroglou & Cohen, 2013; Shiah et al., 2016; Vellem, 2014).

#### Toward optimal levels of various facets of subjective well-being

Psychological science needs to pay more attention to the study of optimal levels not only of happiness but also other components of subjective well-being (see also Oishi et al., 2007). Our study has documented that people across cultures idealize quite different levels of happiness. It seems plausible that across cultures other components of subjective well-being are idealized to various degrees. Data from the World Values Survey (2016) document that religiosity is attributed low importance in secularized WEIRD cultures but top importance in the Middle East, Africa, and Latin America, with an ICC reaching .50 (50% of variance in attributing importance to religion can be explained by the culture level of the analysis).

#### Toward uncovering the consequences of individual-culture (in)congruence in ideals of subjective well-being

The person–environment fit hypothesis states that individuals are better adjusted when their individual attributes are congruent with their proximal and more distal environments (Kristof-Brown et al., 2005). Studies are needed to test a person–culture match in ideal and actual types of various types of subjective well-being. Being moderately happy in a culture governed by the happiness-maximization principle may be a more challenging task than in cultures creating less pressure for happiness. Endorsing a spiritual life may carry different consequences in cultures with a relatively low recognition of spirituality compared with cultures illuminating spirituality (e.g., being atheist in secularized Europe is different from being atheist in countries referring to one or more gods in their constitution). The same is true for being nonreligious in a religious society, and vice versa.

#### Targeting moderate levels of happiness

Taking into consideration that providing high levels of happiness to over seven billion people seems impossible without exceeding critical environmental boundaries (O’Neill et al., 2018), psychological science may need to study how to encourage—mainly WEIRD—people to be more content with moderate levels of happiness. Buddhist thought, for instance, illuminates this reorientation by considering all sources of desire, including happiness, as sources of suffering. Equally, concepts of harmony could provide insights for enhancing balanced self and activity ideals by focusing on maintaining a harmonious life. This consideration can encourage a reflexive stance toward happiness to depart from a pursuit of happiness maximization at any cost and consider instead “a good enough level of happiness.” The second wave of positive psychology (Armstrong et al., 2019; Cohen & Bai, 2019; Ivtzan et al., 2016; Lomas & Ivtzan, 2016; Wang et al., 2016) represents an example of a research program that seeks to supersede WEIRD constraints by including influences from Eastern traditions (dialectical relationship of positive and negative experiences, transcendence of the ego, wisdom, meaning), as heralded by the multicultural study of Chinese values (Chinese Culture Connection, 1987).

### Implications for policymaking

Our work has implications for the policy level as well. A nuanced concept of subjective well-being may inform the development of more diverse measures for assessing good life at the country level (Fabian & Pykett, 2022).

#### Cross-country comparisons of happiness need to acknowledge ideal levels of happiness

International governing bodies seem to formulate policies with the assumption that people in all nations wish to increase their happiness to the same highest levels. For example, the OECD recently adopted personal life satisfaction as the ultimate dependent variable in its cross-national educational comparisons. Because Japanese students reported lower life satisfaction than students in other countries, the OECD instructed the Japanese government to address this issue (Rappleye et al., 2020). Japanese students reported above midpoint happiness, and the OECD experts did not consider that happiness may be idealized in Japan less than in other countries. If international governing bodies (such as the OECD) want to inform policies in a culturally sensitive way (Krys et al., 2020), they may need to learn more about ideal levels of happiness and their interaction with actual levels of happiness, both for cultures and for their individual members. Without such due diligence, policy recommendations may be biased toward specific cultural values—values of WEIRD societies.

#### Culturally sensitive national accounts of well-being

According to the OECD, as of 2018, among 13 (mostly WEIRD) countries calculating NAWB (Diener et al., 2015), 12 rely on happiness as a measure of subjective well-being (Durand, 2018). It seems reasonable to expect that NAWB should guide not only happiness-maximization policies but also “improvement of meaningful life” policies. As noted, Oishi and Diener (2014) documented that levels of meaning in life at the society aggregate are negatively associated with average happiness of societies. Operating with additional components and measures of subjective well-being would contribute to more culturally sensitive (Krys et al., 2020), less ethnocentric psychologizing (Christopher & Hickinbottom, 2008) and hopefully more effective policies (Fabian & Pykett, 2022).

The idea of NAWB (Diener et al., 2015) is a milestone on the pathway beyond narrow economic understanding of national subjective well-being. With the current article, we fully support this direction but signal that personal life satisfaction and happiness-related measures of subjective well-being may be WEIRD-themed. As such, instead of escaping from the overall economization of social reality, they may reinforce it under the guise of endorsing happiness because happiness of societies is positively and strongly related to their economic standing, which is not the case for these societies’ sense of meaning (Oishi & Diener, 2014). To become truly national accounts of subjective well-being, researchers may need to understand the cultural diversity of how people conceptualize subjective well-being, and policymakers need to elaborate indicators that will encompass the cultural diversity of subjective well-being concepts. Because there is probably no single “one-size-fits-all” type of subjective well-being, future NAWB will probably have to be more culturally sensitive (Krys et al., 2020). That is, NAWB may need to be adjusted to the ideals regarding subjective well-being prevalent in a given culture, and, as a result, the unit of analysis shall be the level of progress on indigenously calibrated types of subjective well-being.

### Future directions: open questions about happiness maximization

The fact that our findings provide a large picture of subjective well-being does not diminish the importance and implications of happiness and the tendency to maximize it. They should rather be taken as an opportunity to take stock of its nature and examine it in greater depth.

#### Why maximization of happiness and not other instances of subjective well-being?

In light of the Oishi and Diener (2014) findings that societal happiness is negatively correlated with a country-level sense of meaning, it seems plausible that WEIRD societies tend to maximize happiness, but not equally so other components of subjective well-being (at least not a sense of meaning). Future research may need to answer why cultures that emerged in the most human-friendly habitats maximize a specific type of subjective well-being—happiness—and not other good ways to live (see Morris, 1956). It is also worth exploring further whether the findings presented here for the most popular facet of happiness (life satisfaction) are also valid for facets of happiness typical for non-WEIRD cultures (e.g., interdependent happiness; Hitokoto & Uchida, 2015). Given the predominance of life satisfaction as the operationalization of happiness, this question ultimately requires more empirical study.

#### Ideal self versus ought self

The empirical data we used to verify our reasoning was based on the instruction originally proposed by Diener et al. (2000). In that study, participants were asked to respond to life-satisfaction questions by providing ideal answers. It remains unclear, however, whether these instructions activated the *ideal self* or the *ought self* (Higgins, 1987). The ideal self covers attributes people ideally would like (hope, aspire, and wish) to possess, whereas the ought self relates to attributes people believe they should possess (deriving from their sense of duty, obligation, and responsibility). Future studies may need to test whether the analyses presented here cover both types of self, and if not, further studies on the differences are needed.

#### Better understanding of the costs of happiness maximization

A better understating of maximization should include the comprehensive study of its costs. Does living in a society valuing ceiling levels of happiness indeed create pressure on being more than very happy? If yes, then how? May it indeed lead to increased levels of mania or the consumption of psychoactive substances? Our study was correlational only—causality and mechanisms remain to be tested. Our findings show that the associations for substance use fade away when the cultural syndrome of WEIRDness is controlled for. Thus, it is possible that, for some costs, WEIRDness is the actual cause (and happiness maximization is the covariate), whereas for others happiness maximization has direct causal effects. Furthermore, if members of happy societies are reporting a relatively lower sense of meaning (Oishi & Diener, 2014), then how is happiness related to people’s sense of harmony, or of security, or of predictability? Are societies of happy individuals also societies of happy families and societies of happy local communities?

#### Discussion on side effects of all instances of subjective well-being

A further issue regarding maximization would be to understand whether societies in which other subjective well-being components prevail also have side effects if one strives to maximize their levels. For example, it is not known whether the striving to maximize meaning in life or harmony might also have societal disadvantages; it is also imaginable that maximizing spiritual/religious experience may carry societal disadvantages.

#### Beyond eco-environmental sources of happiness maximization

Our work highlights the relationship between happiness maximization in a culture and the eco-environmental conditions in which a culture emerged. However, natural factors represent only part of a culture’s story. Future research should therefore examine the role that social, political, and perhaps especially religious factors play in pursuing happiness or alternative concepts of subjective well-being. Different types of religion (especially monotheistic vs. polytheistic vs. nontheistic religious traditions) can also be associated with idealized levels of happiness or other components of subjective well-being in different ways (Saroglou, 2019). It is also plausible that the degree to which cultures endorse happiness maximization may vary as a function of optimism, perceived control over outcomes, beliefs about prosperity and hardship, and other cultural syndromes that we did not control for (e.g., flexibility monumentalism; Minkov & Kaasa, 2022)—future studies may need to test such alternative explanations.

#### Emotions and subjective well-being

Culture shapes people’s emotional experiences and beliefs about affect (Tsai, 2007; Tsai et al., 2006). People in WEIRD cultures tend to “savor” positive emotions and “avoid” negative emotions (Miyamoto & Ma, 2011). For Confucians, emotional ideals are a bit more complex. Compared with people in WEIRD cultures, Confucians report fewer and desire less high-arousal emotions (e.g., excitement); the reverse is true for low-arousal emotions (e.g., contentment). Confucians value a sense of appropriate balance and moderation between pleasures and pains rather than valuing or expecting a life of maximum pleasure or happiness (Tsai & Park, 2014). Such contrasting emotional “lifestyles” are likely to influence the ideals of subjective well-being.

### Conceptual clarifications and discussion points

#### Is happiness the ultimate subjective well-being outcome?

We propose the position that phenomena such as harmony, spirituality, and meaning—components of subjective well-being in the broad model—do not “merely” serve the final experience of happiness, or at least not universally. Instead, we propose that treating happiness as the ultimate outcome may be more true in WEIRD cultures than elsewhere, and in other cultures other components of subjective well-being may serve as “final” (or at least that happiness occupies a less prominent position in subjective well-being than in WEIRD cultures). Had mainstream psychology emerged from and been predominantly characterized by Buddhist or Confucian (instead of WEIRD) ideals, we might imagine “pure harmony” as the final experience. Thus, although we do not deny that happiness is for many, maybe even most, people the ultimate goal they strive for, we propose that it is not for all, and that such exceptions are not flukes but rather deserving of careful consideration in theories of subjective well-being.

#### Does life satisfaction capture other forms of subjective well-being sufficiently already?

One might argue that measuring life satisfaction may already capture the degree to which people have obtained contributors to subjective well-being, such as spirituality, harmony, and meaning. However, to the best of our knowledge, there is no strong empirical evidence lending support to the reasoning that individuals base their life-satisfaction evaluations on such other well-being concepts. Instead, some evidence casts doubts on the reasoning that life satisfaction covers all forms of subjective well-being. First, research commonly equates life satisfaction with happiness but not with other subjective well-being components (e.g., Diener et al., 2018; Schimmack, 2006). Second, country-level analyses (Oishi & Diener, 2014) based on 132 nation aggregates show that average life satisfaction in countries is negatively associated with the average sense of meaning in countries, which might bring into question the theoretical assumption that life satisfaction can work as a good proxy for other components of subjective well-being.

#### Differentiating well-being antecedents from components

A challenging task in subjective well-being research, and plausibly psychology in general, is to determine where one phenomenon ends and where another begins. In the case of subjective well-being, for example, should positive affect be considered a trigger of it, or is positive affect part of subjective well-being? Likewise, are phenomena such as meaning, harmony, and family happiness components of subjective well-being or potential causes? If subjective well-being is considered to fluctuate over long rather than short periods of time, then its experiential aspects should probably match that, which is likely the case for meaning, spirituality, harmony, and life satisfaction but possibly less so for positive and negative affect.

#### Are people willing to sacrifice happiness for other pursuits?

Both the narrow and broad models of subjective well-being discussed in the current article propose that sacrifices (e.g., forgoing a positive experience) should ultimately promote their subjective well-being. The broad model implies that this sacrifice may even happen, at least theoretically, in terms of happiness; after all, happiness is but one of several components of subjective well-being. For example, the broad model accounts for situations in which people forgo individual happiness in the pursuit of family harmony, provided that family harmony is more relevant to their subjective well-being than their individual happiness. Indeed, research (Lu, 2008; Lu, 2006; Markus & Kitayama, 1991; Uchida & Kitayama, 2009) has shown that well-being ideals can be pursued regardless of whether they contribute to a “feel-good” destination. In addition, Joshanloo and Weijers, (2014) found that for many people across the world, pursuing and expressing happiness is associated with negative properties such as shallowness, selfishness, reduced empathy, and lack of morality.

#### Don’t people want to be happy?

They do. Our data show that 97% of people idealize being happy over being unhappy. Note that we do not claim that people do not want to be happier than they are—our data show that more than 70% of participants indicated their ideal level of happiness to be higher than their actual level of happiness. However, people across cultures idealize different levels of happiness, and the highest levels of happiness tend to be idealized in WEIRD cultures. Rather than denying that people wish to be happy, we propose, on the basis of this cultural and society diversity, that it is important to further investigate factors other than happiness that may, for some, weight strongly toward their subjective well-being. By bringing the notion of fundamental cultural differences in subjective well-being to the fore, we hope to spark much needed open debate and empirical work.

#### Why the term “subjective well-being” and not another term (such as “mental well-being” or “mental health”)?

A perhaps tempting option is to reserve the term “subjective well-being” for the narrow model and instead use another term for the broad model—such as “mental well-being” or “mental health.” Although we appreciate the benefits that this can have for theory distinction, we propose that it is overall preferable to stick to the term “subjective well-being” for the broad model as well because all components in our proposed broad model are indeed subjective. Furthermore, our article is targeted to encourage broadening studies on subjective well-being from a happiness-related model to a model that assumes a flexible, interdependent construction of subjective well-being. Using a different term from subjective well-being may cause the field to continue to focus on subjective well-being through the three-component model. As a consequence, it may cause people to overlook the arguments presented in our article, as these arguments might falsely appear relevant to a different type of well-being instead.

#### Reminder about avoiding the risk of ecological fallacy

It is important to reiterate that most of the evidence discussed in the current article—such as the links between country happiness and depression, substance use, and so on—rest on data that represent aggregates. Specifically, rather than each individual being represented by a single data point, individuals are grouped together into country-wide averages. Such aggregate data can obscure, misrepresent, or even contradict relations that would be found among the individuals that comprise them. We report them because they provide rarely discussed insights into our topic of inquiry and because we find them worthy of further exploration. However, to avoid falling prey to the ecological fallacy, we express caution in interpreting group-level associations as representing the relations between these variables at the level of individuals.

## Concluding Remarks

Cultural factors play a significant role in shaping what people recognize as the good life. Around the world, people are living their lives in a variety of ways regarded as good from their cultural perspective. Ideals of the good life across cultures reflect this diversity. For some time, psychology has acknowledged that ideals vary across people and societies. We propose and demonstrate that ideal levels of happiness also vary across people and societies, and we hope to reinvigorate the discussion on the variety of types of subjective well-being across cultures.

## Supplemental Material

sj-pdf-1-pps-10.1177_17456916231208367 – Supplemental material for Happiness Maximization Is a WEIRD Way of Living Supplemental material, sj-pdf-1-pps-10.1177_17456916231208367 for Happiness Maximization Is a WEIRD Way of Living by Kuba Krys, Olga Kostoula, Wijnand A. P. van Tilburg, Oriana Mosca, J. Hannah Lee, Fridanna Maricchiolo, Aleksandra Kosiarczyk, Agata Kocimska-Bortnowska, Claudio Torres, Hidefumi Hitokoto, Kongmeng Liew, Michael H. Bond, Vivian Miu-Chi Lun, Vivian L. Vignoles, John M. Zelenski, Brian W. Haas, Joonha Park, Christin-Melanie Vauclair, Anna Kwiatkowska, Marta Roczniewska, Nina Witoszek, I.dil Işık, Natasza Kosakowska-Berezecka, Alejandra Domínguez-Espinosa, June Chun Yeung, Maciej Górski, Mladen Adamovic, Isabelle Albert, Vassilis Pavlopoulos, Márta Fülöp, David Sirlopu, Ayu Okvitawanli, Diana Boer, Julien Teyssier, Arina Malyonova, Alin Gavreliuc, Ursula Serdarevich, Charity S. Akotia, Lily Appoh, D. M. Arévalo Mira, Arno Baltin, Patrick Denoux, Carla Sofia Esteves, Vladimer Gamsakhurdia, Ragna B. Garðarsdóttir, David O. Igbokwe, Eric R. Igou, Natalia Kascakova, Lucie Klůzová Kracˇmárová, Nicole Kronberger, Pablo Eduardo Barrientos, Tamara Mohoricć, Elke Murdock, Nur Fariza Mustaffa, Martin Nader, Azar Nadi, Yvette van Osch, Zoran Pavlovicć, Iva Polácˇková Šolcová, Muhammad Rizwan, Vladyslav Romashov, Espen Røysamb, Ruta Sargautyte, Beate Schwarz, Lenka Selecká, Heyla A. Selim, Maria Stogianni, Chien-Ru Sun, Agnieszka Wojtczuk-Turek, Cai Xing and Yukiko Uchida in Perspectives on Psychological Science

## Transparency

*Action Editor:* Mirta Galesic

*Editor:* Interim Editorial Panel

**Data Availability:** We reused data from other publicly available articles. Our recycled macropsychological database is posted at https://osf.io/uw6kd.

## Notes

1. For the following two articles referred to in this article—Oishi and Diener (2014) and Jebb et al. (2018)—we relate findings that were not presented by the authors of these articles as their main findings. We spotted on our own these particularly interesting patterns of findings. Oishi and Diener (2014) reported, as a secondary finding, that residents of happy countries report a lower sense of meaning overall (*r* = −.33; whereas Oishi and Diener discussed in their article the negative association between GDP per capita and meaning). Jebb et al. (2018) reported as part of secondary findings that across cultures satiation points differ in terms of happiness level (whereas they predominantly discussed differences of satiation points in terms of the income level in main analyses). To spot what we refer to, our readers may need to reread these two articles through the lenses of the reasoning presented here.

2. The cool-water condition is a specific climatic configuration that combines periodically frosty winters with mildly warm summers under the ubiquitous accessibility of fresh water. The cool-water index (Welzel, 2013; after Gallup et al., 2010) combines data on (a) the average annual temperature (inverted), (b) rainfall continuity across the seasons, and (c) the abundance of ice-free waterways on a society’s territory.

3. We also tested the reasoning that (a) a consumption-oriented lifestyle can be related to happiness maximization and manifest itself in the magnitude of the ecological footprint caused by people striving for happiness maximization, and that (b) living in a culture characterized by personal happiness maximization may be related to lessening efforts toward nurturing and sustaining long-lasting relationships that no longer bring high levels of happiness (Wojciszke, 2002) and correlate with society-level averages in divorce. However, the data we analyzed on ecological footprint and divorce rates presented a rather complex picture. Accordingly, we present these findings in the Supplemental Material available online.

4. We do not have access to individual-level data from the Diener et al. data set to enable a similar illustration.
