# Supplementary material for: Happiness Maximization Is a WEIRD Way of Living
Source: Perspect Psychol Sci. 2024 Feb 13;20(5):874–902. doi: 10.1177/17456916231208367 (PMC12408936; doi:10.1177/17456916231208367)
Supplement: sj-pdf-1-pps-10.1177_17456916231208367 – Supplemental material for Happiness Maximization Is a WEIRD Way of Living [file sj-pdf-1-pps-10.1177_17456916231208367.pdf]

Supplementary Online Materials
to the article entitled:

Happiness Maximization Is a WEIRD Way of Life

Kuba Krys, Olga Kostoula, Wijnand A. P. van Tilburg, Oriana Mosca, J. Hannah Lee, Fridanna Maricchiolo, Aleksandra Kosiarczyk, Agata Kocimska-Zych, Claudio Torres, Hidefumi Hitokoto, Kongmeng Liew, Michael H. Bond, Vivian Miu-Chi Lun, Vivian L. Vignoles, John M. Zelenski, Brian W. Haas, Joonha Park, Christin-Melanie Vauclair, Anna Kwiatkowska, Marta Roczniewska, Nina Witoszek, İdil Işık, Natasza Kosakowska-Berezecka, Alejandra Domínguez-Espinosa, June Chun Yeung, Maciej Górski, Mladen Adamovic, Isabelle Albert, Vassilis Pavlopoulos, Márta Fülöp, David Sirlopu, Ayu Okvitawanli, Diana Boer, Julien Teyssier, Arina Malyonova, Alin Gavreliuc, Ursula Serdarevich, Charity S. Akotia, Lily Appoh, Arévalo Mira D.M., Arno Baltin, Patrick Denoux, Carla Sofia Esteves, Vladimer Gamsakhurdia, Ragna B. Garðarsdóttir, David O. Igbokwe, Eric R. Igou, Natalia Kascakova, Lucie Klůzová Kračmárová, Nicole Kronberger, Pablo Eduardo Barrientos, Tamara Mohorić, Elke Murdock, Nur Fariza Mustaffa, Martin Nader, Azar Nadi, Yvette van Osch, Zoran Pavlović, Iva Poláčková Šolcová, Muhammad Rizwan, Vladyslav Romashov, Espen Røysamb, Ruta Sargautyte, Beate Schwarz, Lenka Selecká, Heyla A. Selim, Maria Stogianni, Chien-Ru Sun, Agnieszka Wojtczuk-Turek, Cai Xing, & Yukiko Uchida

corresponding authors:
Kuba Krys: Institute of Psychology, Polish Academy of Sciences, Jaracza 1, Warsaw, 00-378, Poland; e-mail: kuba@krys.pl
Olga Kostoula: Institute of Psychology, Johannes Kepler University Linz, Altenberger St. 69, 4040 Linz, Austria, e-mail: olga.kostoula@jku.at.
Wijnand A. P. van Tilburg: Department of Psychology, University of Essex, Wivenhoe Park, Valley Rd, Colchester CO4 3SQ, UK, email: wijnand.vantilburg@essex.ac.uk

Table of contents:

1. *Latin American Versus WEIRD Societies* 2
2. *Side Effects of Happiness Maximization: DRUGS* 5
3. *Side Effects of Happiness Maximization: ALCOHOL* 6
4. *Side Effects of Happiness Maximization: ECOLOGY* 7
5. *Side Effects of Happiness Maximization: DIVORCE* 9
6. *Side Effects of Happiness Maximization: MANIA* 11

1. Latin American Versus WEIRD Societies

When it comes to country-level happiness, both North-Western European and Latin American societies tend to occupy top ranks (e.g., Minkov, 2009; Veenhoven, 2016). However, while North-Western European societies tend to be WEIRD, Latin American societies tend not to. A comparison between the ideal happiness amongst these sets offers, accordingly, a particularly interesting opportunity to lend additional support to our reasoning. After all, their difference in WEIRD factor status seems independent of actual happiness levels (different from the general trends analyzed in the main text). For this reason, we here zeroed in on the contrast between Latin American societies versus North-Western European ones in the two datasets.

Indeed, Latin America and North-Western Europe scored comparatively high on average actual happiness, and did not differ significantly from each other in neither of the analysed datasets, $ts < .64$; $ps > .53$ (Table S6). We found that the average ideal happiness was significantly lower in Latin American countries then in than North-Western European ones in Krys et al. (2020) dataset, $t[15] = 3.71$; $p = .002$; $d = 1.83$, but not in the Diener et al. (2000) dataset, $t[8] = .18$; $p = .86$; $d = .11$; when we analysed both datasets combined into one dataset Latin Americans idealized happiness less than North-Western Europeans, $t[19] = 2.81$; $p = .011$; $d = 1.23$ (Table S1). Thus, direct comparison of Latin American and WEIRD societies delivers partial support (Krys et al. data and combined datasets, but not Diener et al. dataset) to our theorising that the ideal levels of happiness vary systematically depending on their WEIRD status, even if actual happiness levels appear no different. These findings further emphasize that idealization of high happiness as superordinate facet of subjective well-being is more cross-culturally variable then typically theorized.

Table S1

Ideal and Actual Levels of Happiness Across Cultural Macro-Regions

		Happiness			
		Ideal		Actual	
Region	*N*	*M*	*(SD)*	*M*	*(SD)*
**DIENER ET AL. (2000)**					
North-Western Europe	5	.78	.22	1.13	.41
Southern Europe	4	.84	.33	.02	.46
Central Europe	4	.39	.37	-.10	1.14
Latin America	5	.74	.51	.92	.93
ACNU	2	.94	.59	.70	.20
MENA	5	-.27	.96	-.29	.83
Post-Soviet Countries	-	-	-	-	-
Confucian Asia	6	-.72	1.26	-1.09	.92
Southern Asia & Oceania	5	-.82	.44	.07	.48
Sub-Saharan Africa	5	-.91	.89	-.73	.63
Whole dataset	41	.00	1.00	.00	1.00
**KRYS ET AL. (2020)**					
North-Western Europe	10	1.04	.42	.76	.50
Southern Europe	3	.11	.32	.22	.33
Central Europe	9	.66	.86	.46	1.02
Latin America	7	.02	.71	.60	.57
ACNU	3	-.64	.91	-.47	.59
MENA	4	-.35	.52	-.22	.57
Post-Soviet Countries	3	-.44	.45	-1.18	.51
Confucian Asia	5	-1.18	.60	-1.41	.71
Southern Asia & Oceania	7	-1.11	.72	-.33	.74
Sub-Saharan Africa	3	-1.44	.28	-1.35	.77
Whole dataset	49	.00	1.00	.00	1.00
**BOTH DATASETS COMBINED**					
North-Western Europe	12	.93	.31	.83	.46
Southern Europe	4	.62	.48	.13	.36
Central Europe	10	.53	.77	.30	.97
Latin America	9	.43	.51	.82	.58
ACNU	3	-.02	.17	-.01	.31
MENA	7	-.25	.81	-.24	.55
Post-Soviet Countries	3	-.44	.45	-1.18	.51
Confucian Asia	6	-.82	.76	-1.11	.69
Southern Asia & Oceania	7	-.96	.53	-.11	.62
Sub-Saharan Africa	5	-1.05	.88	-.90	.58
Whole dataset	66	.04	.92	.06	.91

Note. Southern Europe: Portugal, Spain, Italy, and Greece (these Southern European countries are not recognized as individualistic, thus, we created separate cultural cluster from them); Latin America covers all American countries apart from Canada and the USA; ACNU: Australia, Canada, New Zealand, and the USA; MENA – Middle East and North Africa; Post-Soviet countries cover all former USSR countries excluding Lithuania, Latvia and Estonia that currently belong to the European Union and that are included into Central Europe

Also, we paid particular attention to the contrast of existential pressures between North-Western European societies versus Latin American Societies, given their matched *actual* happiness levels, but different *ideal* levels of happiness. North-Western Europe ranked among the top least existentially pressured societies in terms of our ecology indexes, whereas Latin American societies ranked lower (Figure 2 of the main text). Indeed, Latin American ecologies scored lower than North-Western European societies in terms of the cool water index ($M_{\text{Latin America}} = .37$, $SD_{\text{Latin America}} = .13$, $M_{\text{North-Western Europe}} = .80$, $SD_{\text{North-Western Europe}} = .11$, $t[38] = 10.53$, $p <. 001$, $d = 3.53$), pathogen security ($M_{\text{Latin America}} = .43$, $SD_{\text{Latin America}} = .12$, $M_{\text{North-Western Europe}} = .77$, $SD_{\text{North-Western Europe}} = .08$, $t[41] = 9.24$, $p <. 001$, $d = 3.27$), and natural disasters security ($M_{\text{Latin America}} = 5.57$, $SD_{\text{Latin America}} = 6.80$, $M_{\text{North-Western Europe}} = 12.09$, $SD_{\text{North-Western Europe}} = 1.44$, $t[42] = 3.53$, $p = .001$, $d = 1.58$).

Despite being high on actual happiness, Latin Americans thus live in relatively harsher ecological niches than North-Western Europeans, and at the same time they idealize happiness moderately only. This may lend indicate that whether, or not, societies idealize high levels of happiness may relate to the ecology they occupy.

2. Side Effects of Happiness Maximization: DRUGS

Table S2

Side-Effects - Drugs: Drug Use and Abuse and Ideal Level of Personal Life Satisfaction

		cannabis	cocaine	ecstasy	amphetamines	opiates	tranquillizers	**Drugs – meta factor**	opioids	prescription opioids	prescription stimulants	**Drugs – meta factor broader**
	DIENER ET AL.											
(1)	zero-order correlation	.35^{+}	.41*	.32	.02	.17	.23	**.37***	.03	.48	.60	**.36***
(2)	pc: actual LS	.21	.33^{+}	.34^{+}	-.10	-.02	.11	**.20**	-.01	.26	.79^{+}	**.14**
(3)	pc: WEIRDness	.30	.26	.11	-.07	.17	.12	**.21**	-.07	.28	.43	**.21**
(4)	*N* (number of countries)	30	29	25	28	29	19	**34**	12	11	6	**34**
	KRYS ET AL.											
(1)	zero-order correlation	.17	-.07	.00	-.03	-.07	.49**	**.14**	-.28	.15	-.65^{+}	**.13**
(2)	pc: actual LS	.16	.00	.16^{+}	.17^{+}	.46**	.42*	**.34***	-.07	.10	-.28	**.31***
(3)	pc: WEIRDness	.00	-.43**	-.20	-.14	-.04	.45*	**-.10**	-.33	.08	.-75*	**-.09**
(4)	*N* (number of countries)	45	41	40	42	39	29	**46**	17	18	8	**46**
	DATASETS COMBINED											
(1)	zero-order correlation	.31*	.18	.14	-.04	-.02	.43*	**.28***	-.20	.31	-.20	**.27***
(2)	pc: actual LS	.25^{+}	.20	.27^{+}	.00	.26^{+}	.34^{+}	**.33***	-.15	.15	.12	**.30***
(3)	pc: WEIRDness	.15	-.11	-.11	-.14	-.03	.38*	**.01**	-.27	.23	-.30	**.04**
(4)	*N* (number of countries)	56	52	49	53	51	33	**60**	23	21	10	**60**

Note: VIFs < 1.83; pc = partial correlation: controlling for actual life satisfaction (actual LS) and WEIRDness. Number of analyzed countries varies depending on the country-overlap between datasets; Drugs metafactor – we standardized scores within the first six datasets on drugs (they contain more countries than the remaining three datasets), and then for each country we calculated mean from available standardized scores; Drugs metafactor broader – procedure for drugs metafactor run for all nine datasets on drugs. We present also non-significant findings to document which datasets we analyzed. The data on drugs are taken from the United Nations, Office on Drugs and Crime. (2019). Drug use and prevalence [Data set]. Retrieved from httpps://dataunodc.un.org/dp-drug-use-prevalence

3. Side Effects of Happiness Maximization: ALCOHOL

Table S3

Side-Effects - Alcohol: Alcohol Use and Abuse and Ideal Level of Personal Life Satisfaction

		alc. year percentage	daily gram	abstainers lifetime (reversed)	heavy drinking episodes (age standardized)	alc. consumers past 12 months	**alcohol – meta factor**
	DIENER ET AL.						
(1)	zero-order correlation	.40*	-.17	.19	.02	.29+	**.27**
(2)	partial correlation: actual life satisfaction	.36*	.02	.12	.11	.35*	**.34***
(3)	partial correlation: WEIRDness	-.08	-.01	.10	.19	-.21	**-.10**
(4)	*N* (number of countries)	35	36	35	36	35	**36**
	KRYS ET AL.						
(1)	zero-order correlation	.46***	.02	.20	.29*	.52***	**.49*****
(2)	partial correlation: actual life satisfaction	.52***	.24	.17	.30*	.61***	**.60*****
(3)	partial correlation: WEIRDness	.15	.08	.21	.13	.26+	**.29+**
(4)	*N* (number of countries)	46	46	46	46	46	**46**
	DATASETS COMBINED						
(1)	zero-order correlation	.48***	-.09	.21+	.19	.44***	**.41****
(2)	partial correlation: actual life satisfaction	.48***	.13	.16	.22+	.48***	**.47*****
(3)	partial correlation: WEIRDness	.05	.00	.12	-.01	-.01	**.07**
(4)	*N* (number of countries)	60	61	60	61	60	**61**

Note: VIFs < 1.56; Number of analyzed countries varies depending on the country-overlap between datasets. Alcohol metafactor – we standardized scores within each of the five datasets on alcohol, and then for each country we calculated mean from available standardized scores. We present also non-significant findings to document which datasets we analyzed. The data on alcohol are taken from WHO, Global Health Observatory Data Repository. (2019). Global Information System on Alcohol and Health [Data set] Retrieved from https://www.who.int/data/gho/data/themes/global-information-system-on-alcohol-and-health

4. Side Effects of Happiness Maximization: ECOLOGY

Table S4
Side-Effects - Ecology: Correlation between Ecological Footprint of Consumption and Ideal Level of Personal Life Satisfaction

		ecological footprint consumption
	DIENER ET AL.	
(1)	zero-order correlation	.25
(2)	partial correlation - controlling for:	
	actual personal life satisfaction	.22
	GDP *per capita* (log transformed)	-.44**
	both GDP *per capita* and actual life satisfaction	-.35*
(3)	*N* (number of countries)	36
	KRYS ET AL.	
(1)	zero-order correlation	.23+
(2)	partial correlation - controlling for:	
	actual personal life satisfaction	.27+
	GDP *per capita* (log transformed)	-.14
	both GDP *per capita* and actual life satisfaction	.03
(3)	*N* (number of countries)	45
	DATASETS COMBINED	
(1)	zero-order correlation	.23+
(2)	partial correlation - controlling for:	
	actual personal life satisfaction	.21
	GDP *per capita* (log transformed)	-.38**
	both GDP *per capita* and actual life satisfaction	-.25+
(3)	*N* (number of countries)	61

Note: VIFs < 4.61; Number of analyzed countries varies depending on the country-overlap between datasets.

We found a weak association between ecological footprint of consumption and happiness maximization (with statistical significance reaching the level of tendency and falling below it when actual life satisfaction was controlled for). Furthermore, because the ecological footprint is unequally distributed, with residents of high-income countries placing a disproportionate pressure on nature (WWF, 2020), in our analyses we additionally controlled for GDP *per capita*. We found that when GDP *per capita* is controlled for,

happiness maximization is a negative predictor of ecological footprint of consumption (see above Table). Thus, the findings on ecological footprint do not lend support to our reasoning that a lifestyle of “happiness maximization” may be significantly related to environmental pressure. In contrast to our predictions, when economic indicators are controlled for, “happiness maximization” may be related to lower environmental pressure.

5. Side Effects of Happiness Maximization: DIVORCE

Table S5

Side-Effects – Divorce Rate

		Divorce rates
	DIENER ET AL.	
(1)	zero-order correlation	.15
	partial correlation - controlling for:	
(2)	*actual* personal life satisfaction	.08
(3)	marriage rate	.32
(4)	WEIRDness	-.14
(5)	both marriage rate and actual life satisfaction	.11
(6)	all three factors (rows: 3, 4, 5) at the same time	-.15
(7)	*N* (number of countries)	28
	KRYS ET AL.	
(1)	zero-order correlation	.11
	partial correlation - controlling for:	
(2)	*actual* personal life satisfaction	.53***
(3)	marriage rate	.19
(4)	WEIRDness	-.03
(5)	both marriage rate and actual life satisfaction	.50**
(6)	all three factors (rows: 3, 4, 5) at the same time	.36*
(7)	*N* (number of countries)	40
	DATASETS COMBINED	
(1)	zero-order correlation	.12
	partial correlation - controlling for:	
(2)	*actual* personal life satisfaction	.30*
(3)	marriage rate	.24+
(4)	WEIRDness	-.07
(5)	both marriage rate and actual life satisfaction	.30*
(6)	all three factors (rows: 3, 4, 5) at the same time	.13
(7)	*N* (number of countries)	51

Note: VIFs < 2.68; Number of analyzed countries varies depending on the country-overlap between datasets. We excluded from our analysis data from Guam (populated by less than 200,000 people) for which we found extremely high level of divorce rate (over 6.5 SD above average). The data on divorce are taken from United Nations, Department of Economic and Social Affairs, Statistics Division. (2020). Population by marital status, age, sex, and urban/rural residence [Data set]. Retrieved from data.un.org/Data.aspx?d=POP&f=tableCode%3a23

Living in a culture characterized by personal happiness maximization may also be related to lessening efforts towards nurturing and sustaining long-lasting relationships that no longer bring high levels of happiness (Wojciszke, 2002) and lead to life-decisions like divorce. In this domain, too, we found at the country-level of analysis that “happiness maximization” calculated for both datasets combined predicts the divorce rate, after controlling for marriage rate, after controlling for actual happiness, and after controlling for both (although findings lend only partial support when both datasets are analysed separately - see supplementary online material), but not after controlling for the cultural syndrome of WEIRDness. Additionally, another important confounder can play the role in this association – gender egalitarian policies. Therefore, our analyses show partial only support to the reasoning, and demand further research.

6. Side Effects of Happiness Maximization: MANIA

Table S6

Side-Effects – Mania Prevalence

		bi-polar disorder prevalence
	DIENER ET AL.	
(1)	zero-order correlation	.62***
(2)	partial correlation - controlling for actual life satisfaction	.48**
(3)	partial correlation - controlling for WEIRDness	.44**
(4)	*N* (number of countries)	37
	KRYS ET AL	
(1)	zero-order correlation	.34*
(2)	partial correlation - controlling for actual life satisfaction	.16
(3)	partial correlation - controlling for WEIRDness	.12
(4)	*N* (number of countries)	46
	DATASETS COMBINED	
(1)	zero-order correlation	.55***
(2)	partial correlation - controlling for actual life satisfaction	.40**
(3)	partial correlation - controlling for WEIRDness	.33**
(4)	*N* (number of countries)	62

Note: VIFs < 3.00; Number of analyzed countries varies depending on the country-overlap between datasets. The data on mania are taken from Our World in Data, Mental Health, Bipolar Disorder. (2019). Bipolar disorder [Data Set]. Retrieved from https://ourworldindata.org/mental-health#bipolar-disorder
